# Oncogenic KRAS/ERK/JUNB signaling suppresses differentiation regulator *GATA6* in pancreatic cancer

**DOI:** 10.1172/JCI191370

**Published:** 2025-12-02

**Authors:** Zheng Zhong, Xinang Cao, Pei-Ju Liao, Raman Sethi, Jeffrey A. Klomp, Clint A. Stalnecker, Jinmiao Chen, Yue Wan, Channing J. Der, David M. Virshup

**Affiliations:** 1 Program in Cancer and Stem Cell Biology, Duke-NUS Medical School, Singapore.; 2 Genome Institute of Singapore and; 3 Bioinformatics Institute, Agency for Science, Technology and Research (A*STAR), Singapore.; 4 Lineberger Comprehensive Cancer Center and; 5 Department of Pharmacology, University of North Carolina at Chapel Hill, Chapel Hill, North Carolina, USA.; 6 Centre for Computational Biology, Duke-NUS Medical School, Singapore.; 7 Department of Pediatrics, Duke University, Durham, North Carolina, USA.

**Keywords:** Cell biology, Oncology, Cancer, Signal transduction

## Abstract

*GATA6* is a master regulator of differentiation in the pancreas, and its expression levels determine the 2 main molecular subtypes of pancreatic cancer. High GATA6 levels contribute to the classical pancreatic cancer subtype, which is associated with a higher degree of tumor differentiation and better disease prognosis. However, why *GATA6* expression varies across pancreatic cancers and what regulates *GATA6* expression remain elusive. Here, we report that oncogenic KRAS-activated ERK signaling suppresses *GATA6* transcription in pancreatic cancers. *GATA6* mRNA levels inversely correlated with KRAS/ERK activity in pancreatic tumors. A genome-wide CRISPR screen in a *GATA6*-*EGFP* reporter knockin cell line identified JUNB as the ERK-regulated transcriptional repressor for *GATA6*. Active ERK stabilized JUNB protein, while KRAS/ERK inhibition led to ubiquitin-independent proteasomal degradation of JUNB and increased transcription of *GATA6*. Upregulation of GATA6 enhanced chemosensitivity of pancreatic cancer cells, and KRAS/ERK inhibitors synergized with chemotherapy in a GATA6-dependent manner. Our study identifies how oncogenic KRAS/ERK signaling suppresses *GATA6* to cause dedifferentiation in pancreatic cancer. Combining KRAS/ERK inhibitors with standard-of-care chemotherapies could be a promising therapeutic strategy for treating pancreatic cancers.

## Abstract 2

Hyperactivated KRAS/ERK stabilizes the transcriptional repressor JUNB, which suppresses the expression of GATA6, the master regulator of differentiation and chemosensitivity in pancreatic cancers.

## Graphical abstract

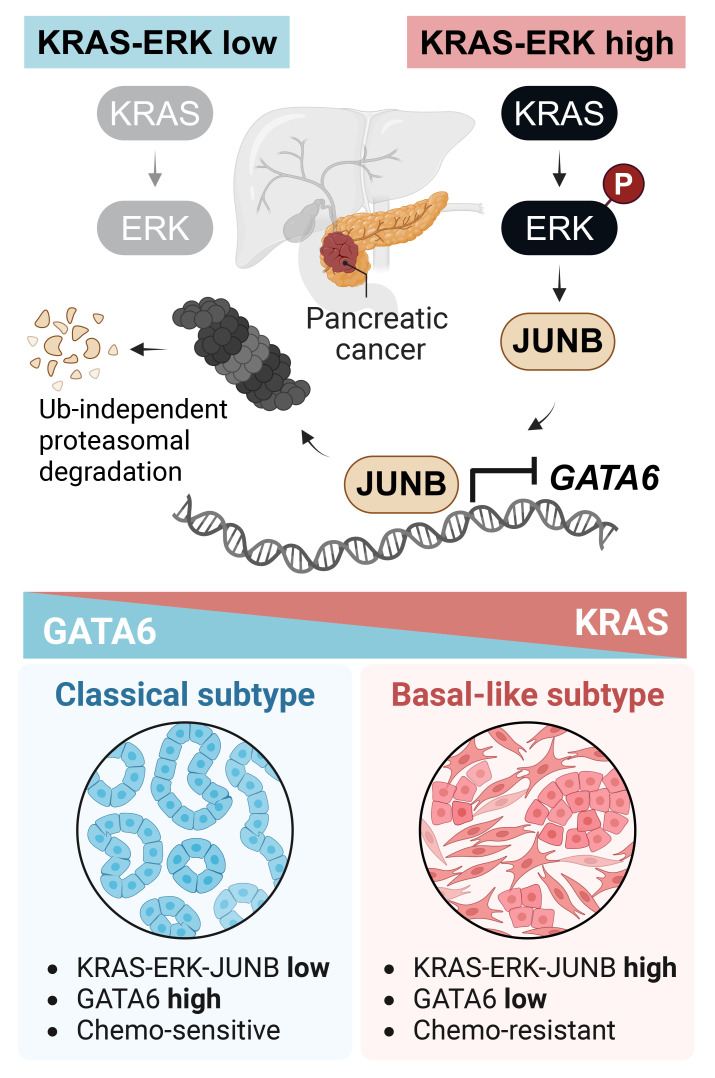

## Introduction

Pancreatic cancer (pancreatic ductal adenocarcinoma [PDAC]) is one of the most lethal cancer types worldwide (1, 2). Most pancreatic cancers are diagnosed at advanced stages, and the treatment for these patients largely relies on conventional chemoradiotherapies that provide only modest survival benefits (3).

Transcriptomic analyses of pancreatic tumors have revealed 2 major molecular subtypes that are associated with distinct disease prognosis: the classical and basal-like subtypes (4, 5). The classical subtype pancreatic cancers feature better tumor differentiation, less metastases, superior therapy response, and longer patient survival (5, 6). Notably, the expression level of the *GATA6* gene is sufficient to efficiently distinguish these 2 subtypes: high expression of *GATA6* correlates well with the classical subtype, making GATA6 a valuable marker that could be incorporated into routine histological examinations for pancreatic cancers (5, 7).

Importantly, the classical and basal-like states of pancreatic cancer are interconvertible, and GATA6, besides being a biomarker, is functionally involved in the cancer state determination. GATA6 is a zinc-finger transcription factor, a member of a protein family so named for their DNA sequence binding specificity (8). Members of the GATA family are lineage-specific differentiation regulators, and GATA6 regulates the development of heart and several endodermal tissues, including pancreas (9–11). In pancreatic tumors, manipulating the expression levels of GATA6 led to phenotypic switching between the 2 cancer subtypes, affecting both tumor differentiation status and drug sensitivities (12–14). These findings establish GATA6 as a master regulator of differentiation in pancreatic cancer and suggest that boosting GATA6 expression may improve patient outcomes. However, why GATA6 expression is heterogeneous and how it is regulated remain elusive.

Nearly all pancreatic cancers are driven by oncogenic *KRAS* mutations, while variations in *KRAS* mutant types and copy numbers, together with other genetic and nongenetic events, contribute to the inter- and intratumor heterogeneity (15–17). Interestingly, KRAS inhibition in pancreatic tumors led to a rapid enrichment of cancer cells with the GATA6-high classical state (18, 19), suggesting that oncogenic KRAS signaling could be involved in the determination of pancreatic cancer state. However, how KRAS might influence GATA6 and cancer differentiation is not well understood.

We previously identified rare p300 mutations that silenced *GATA6* expression, driving phenotype switching and drug resistance in pancreatic cancers (14). Looking for more common regulators of GATA6 expression, here, we report that the hyperactivated KRAS/ERK axis suppresses the transcription of *GATA6*. Through a genome-wide CRISPR screen, we identified the transcriptional repressor JUNB as the major inhibitor of *GATA6* expression. Active ERK stabilized JUNB protein, while KRAS/ERK inhibition led to ubiquitin-independent proteasomal degradation of JUNB and hence promoted *GATA6* expression. Upregulation of GATA6 enhanced chemosensitivity of pancreatic cancer cells, leading to synergy between KRAS/ERK inhibitors and conventional chemotherapy. In summary, our study links KRAS/ERK signaling, the major oncogenic driver, to GATA6, the master regulator of differentiation in pancreatic cancers. These findings provide a molecular rationale for combining KRAS/ERK inhibitors with standard-of-care chemotherapies for treating pancreatic cancers.

## Results

### Inhibiting the KRAS/MEK/ERK axis boosts GATA6 expression in pancreatic cancer.

**Figure 1:**
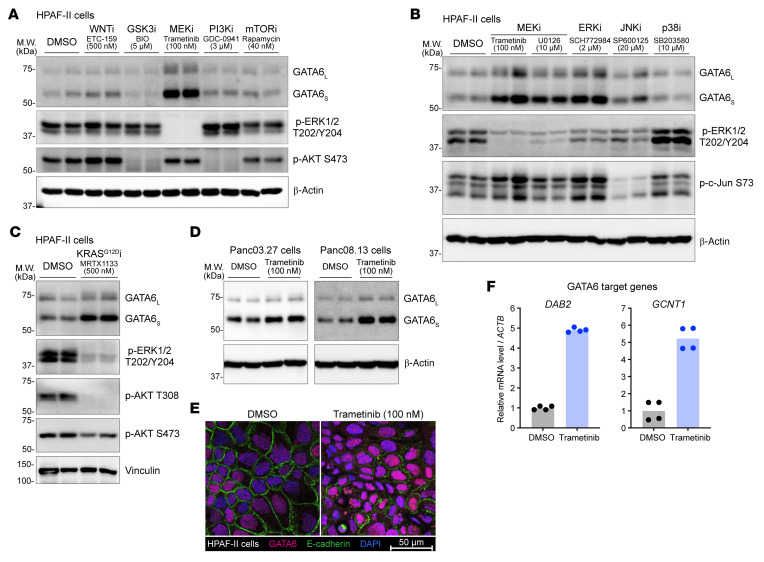
Inhibiting the oncogenic KRAS/MEK/ERK axis upregulates GATA6 expression in pancreatic cancer. (**A**–**C**) Inhibiting the KRAS/MEK/ERK axis increased GATA6 protein abundance. HPAF-II cells were treated with small-molecule inhibitors (e.g., WNT inhibitor [WNTi]) at the indicated concentrations for 24 h, followed by Western blot analysis. GATA6 has 2 isoforms due to 2 translational start sites, generating GATA6_L_ and GATA6_S_, respectively. β-Actin or vinculin was used as loading control. Each lane represents a biological replicate. *n* = 2 biological replicates/condition. (**D**) Panc03.27 and Panc08.13 cells were treated with MEK inhibitor trametinib for 24 h, followed by Western blot analysis. *n* = 2 biological replicates/condition. (**E**) MEK inhibition led to nuclear accumulation of GATA6. HPAF-II cells were treated with trametinib for 24 h, followed by immunofluorescence staining. E-cadherin and DAPI staining marked cell boundaries and nuclei, respectively. Scale bar: 50 μm. (**F**) MEK inhibition upregulated the expression of GATA6 target genes. HPAF-II cells were treated with trametinib (100 nM) for 24 h, followed by RT-qPCR analysis. *n* = 2 biological replicates × 2 technical replicates/condition.

GATA6 protein abundance varies across pancreatic tumors and can change during cancer progression, suggesting dynamic regulation of its expression. To identify the upstream signaling that regulates GATA6 expression, we treated HPAF-II cells, a pancreatic cancer cell line driven by oncogenic KRAS^G12D^ and hypersensitivity to WNTs (20), with inhibitors targeting key nodes of these pathways. We found that MEK inhibition by trametinib dramatically increased GATA6 protein abundance (Figure 1A). Active MEK mainly activates the downstream ERK but may also crosstalk with related MAPK cascades (21), e.g., JNK and p38. To determine the effectors downstream of MEK that regulate GATA6 and rule out the possibility of off-target effects, we next treated the cells with a structurally unrelated MEK inhibitor as well as inhibitors specifically targeting the ERK, JNK, and p38 kinases. Only inhibitors of MEK and ERK potently increased GATA6 abundance (Figure 1B). Oncogenic KRAS mutations activate the MEK/ERK axis in pancreatic cancers. Consistent with this, a specific KRAS^G12D^ inhibitor also boosted GATA6 abundance, similar to the effect of the MEK and ERK inhibitors (Figure 1C). Furthermore, we observed the same phenotype in 2 additional pancreatic cancer cell lines with mutant KRAS but without WNT hypersensitivity (Figure 1D). Thus, the oncogenic KRAS-activated MEK/ERK axis can suppress GATA6 expression in pancreatic cancer cells.

GATA6 protein can be phosphorylated by kinases including ERK, and site-specific phosphorylation of GATA6 has been reported to be important for its nuclear localization and/or transcriptional activity (22, 23). Our data revealed that inhibiting the hyperactive MEK/ERK cascade led to marked GATA6 protein accumulation. We next tested if the accumulated GATA6 protein was still functional. Immunofluorescence staining confirmed the nuclear accumulation of GATA6 upon MEK/ERK inhibition (Figure 1E); importantly, this was accompanied by substantially increased expression of established GATA6 target genes *DAB2* and *GCNT1* (24, 25) (Figure 1F). These results indicate that MEK/ERK inhibition potently promotes GATA6 signaling by increasing the abundance of nuclear and fully functional protein.

GATA4 is closely related to and has similar lineage specificity as GATA6 and may cooperate with GATA6 in regulating pancreatic cancer subtypes (26). However, MEK inhibition did not upregulate GATA4 expression in 2 pancreatic cancer cell lines (Supplemental Figure 1; supplemental material available online with this article; https://doi.org/10.1172/JCI191370DS1), indicating that unlike GATA6, GATA4 is not regulated by KRAS/ERK signaling in pancreatic cancers.

### KRAS/ERK signaling inhibits GATA6 transcription in vivo.

**Figure 2:**
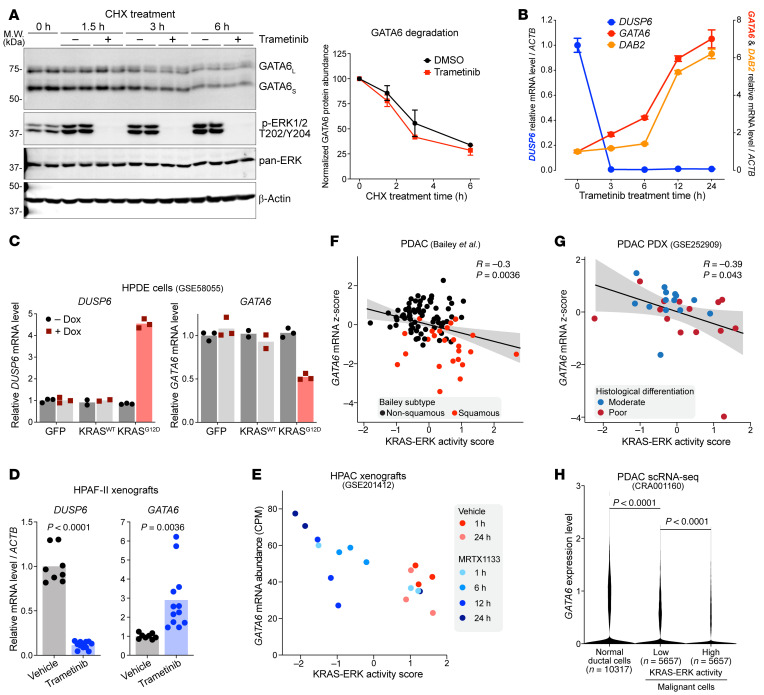
Oncogenic KRAS-activated ERK signaling inhibits *GATA6* transcription. (**A**) MEK inhibition did not stabilize GATA6 protein. HPAF-II cells were treated with 40 μg/mL CHX in the presence or absence of 100 nM trametinib for the indicated time. Quantification (mean ± SD) of GATA6 bands is shown. *n* = 2 biological replicates/condition. (**B**) MEK inhibition upregulated *GATA6* transcript abundance. HPAF-II cells were treated with 100 nM trametinib for the indicated time. *DUSP6* is an ERK target gene, while *DAB2* is a GATA6 target gene. *n* = 2 biological replicates × 2 technical replicates/condition. Data are shown as the mean ± SD. (**C**) Oncogenic activation of KRAS/ERK signaling downregulated *GATA6*. Analysis of data from GSE58055 (27). Human pancreatic duct epithelial (HPDE) cells expressing dox-inducible GFP, WT KRAS, or KRAS^G12D^ mutant were profiled by microarray. (**D** and **E**) KRAS/ERK inhibition upregulated *GATA6* in vivo. (**D**) Mice bearing HPAF-II xenografts were treated with vehicle or trametinib (3 mg/kg) once daily for 3 days, and tumors were harvested 8 h after the last dose for analysis. *n* = 4–6 tumors per condition each analyzed with technical replicates. *P* values of 2-tailed, unpaired *t* test are shown. (**E**) Analysis of data from GSE201412 (28). Mice bearing HPAC xenografts were treated with vehicle or KRAS^G12D^ inhibitor and harvested at the indicated time points. (**F** and **G**) KRAS/ERK activity is inversely correlated with *GATA6* transcript abundance. Analyses of data from references 31 and 32. Each dot represents a human primary pancreatic cancer (**F**) or a PDAC PDX (**G**). Samples were colored based on the Bailey molecular subtype in **F** or histological differentiation status in **G**. The *R* and *P* values of simple linear regression are shown. (**H**) Pancreatic cancer cells with high KRAS/ERK activity showed lower *GATA6* expression (CRA001160) (34). The *P* values of Dunn’s multiple-comparison test are shown.

We next investigated how MEK/ERK inhibition increases GATA6 protein abundance. While in some cases protein phosphorylation regulated protein degradation, MEK/ERK inhibition did not affect GATA6 protein stability (Figure 2A). Instead, *GATA6* transcript abundance increased rapidly upon MEK/ERK inhibition, followed by an increase in expression of the GATA6 target gene *DAB2* (Figure 2B). This indicates that active ERK suppresses *GATA6* transcription. Supporting this, we analyzed a public dataset (27) where doxycycline-inducible (dox-inducible) mutant KRAS^G12D^ was expressed in immortalized human pancreatic duct epithelial cells. There, oncogenic KRAS-activated MEK/ERK signaling, evidenced by the upregulated expression of the ERK target gene *DUSP6*, also clearly downregulated *GATA6* mRNA abundance (Figure 2C).

To determine whether this regulation occurs in vivo, we treated mice bearing HPAF-II subcutaneous xenografts with the MEK inhibitor trametinib. Trametinib-treated tumors had a significant decrease in *DUSP6* expression, confirmed the inhibition of MEK/ERK signaling, and, importantly, had significantly increased *GATA6* mRNA abundance (Figure 2D). In addition, we analyzed an RNA-seq dataset (28) where mice bearing HPAC or AsPC-1 tumors (two *KRAS^G12D^*-mutant human PDACs) were treated with the KRAS^G12D^-specific inhibitor MRTX1133. KRAS inhibition downregulated KRAS/ERK signaling, as scored with a recently established KRAS/ERK-dependent gene expression signature (29) (see Methods). Importantly, in both models, KRAS inhibition upregulated *GATA6* mRNA abundance (Figure 2E and Supplemental Figure 2). Moreover, in a public RNA-seq dataset of murine pancreatic cancer organoids driven by *Kras^G12D^* (30), MEK inhibition also boosted mouse *Gata6* expression (Supplemental Figure 3). Taken together, these results reveal a robust negative regulatory axis where oncogenic KRAS-activated ERK signaling suppresses *GATA6* transcription in pancreatic cancers.

To examine the clinical relevance of our findings, we analyzed the activity of KRAS/ERK signaling and *GATA6* mRNA levels in human pancreatic tumors. Consistent with our model that active ERK suppresses *GATA6* transcription, *GATA6* mRNA levels negatively correlated with KRAS/ERK activity in a cohort of resected human pancreatic tumors (31) (Figure 2F). Pancreatic tumors are rich in stroma. To examining whether this negative correlation between KRAS/ERK activity and *GATA6* expression in bulk RNA-seq resulted from differential gene expression specifically in the cancer cells, we analyzed transcriptomic datasets of patient-derived xenografts (PDXs) in mice where we could focus on cancer cells by specifically analyzing the human transcriptome. In 2 independent cohorts of pancreatic cancer xenografts (32, 33), *GATA6* expression inversely correlated with KRAS/ERK activity in cancer cells (Figure 2G and Supplemental Figure 4A). Finally, scRNA-seq of primary human pancreatic cancers and normal pancreases revealed that malignant cells expressed lower levels of *GATA6* compared with normal ductal cells (34) (Figure 2H), which is consistent with the assumption that almost all these malignant cells harbor *KRAS* mutations. Within the malignant cell population, cells with a higher KRAS/ERK activity score expressed lower levels of *GATA6* (Figure 2H), reflecting the negative regulation on a single-cell level.

Notably, consistent with the model that KRAS/ERK suppresses *GATA6* transcription while GATA6 protein promotes differentiation, tumors of the squamous subtype, the Bailey classification subtype that corresponds to the dedifferentiated *GATA6*-low basal-like subtype, showed significantly higher KRAS/ERK activity compared with nonsquamous tumors in the Bailey cohort (Figure 2F and Supplemental Figure 4B). Similarly, PDAC PDXs with lower KRAS/ERK and higher *GATA6* tended to be better differentiated (Figure 2G). Taken together, these data suggest that the heterogeneous KRAS/ERK signaling is involved in regulating pancreatic cancer differentiation at least partially through suppressing *GATA6*.

### Genome-wide CRISPR screening identifies JUNB as a transcriptional repressor of GATA6.

**Figure 3:**
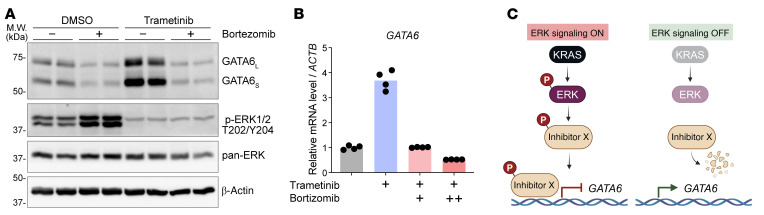
ERK inhibits *GATA6* transcription, potentially by stabilizing certain transcriptional repressor(s). (**A** and **B**) Proteasome inhibition prevented the upregulation of GATA6 by MEK inhibition. (**A**) HPAF-II cells were treated with DMSO or 100 nM trametinib, with or without 10 nM bortezomib for 24 h, followed by Western blot analysis. *n* = 2 biological replicates/condition. (**B**) HPAF-II cells were treated with DMSO or 100 nM trametinib, with or without 5 (+) or 10 (++) nM bortezomib for 24 h, followed by RT-qPCR analysis. *n* = 2 biological replicates × 2 technical replicates/condition. (**C**) A model of how KRAS/ERK might inhibit *GATA6* transcription.

We further investigated the mechanism by which ERK suppresses *GATA6* transcription. While the increase of GATA6 abundance upon MEK/ERK inhibition was not due to decreased GATA6 protein degradation, we made the unexpected finding that blocking the proteasomal degradation pathway by bortezomib caused a marked decrease in GATA6 protein abundance (Figure 3A). More surprisingly, bortezomib completely blocked the effect of MEK/ERK inhibition on GATA6 protein and mRNA levels (Figure 3, A and B). MEK/ERK inhibition upregulated *GATA6* mRNA abundance in as little as 3 h (Figure 2B). This rapid time course suggested that ERK is directly phosphorylating a factor controlling *GATA6* transcription, rather than causing a secondary transcriptional response, e.g., via an indirect and delayed impact on another target gene such as *DAB2*. Given that proteasome inhibition mitigated the effect of MEK/ERK inhibition, we speculated that active ERK phosphorylated and therefore stabilized a transcriptional repressor(s) that then inhibited *GATA6* transcription (Figure 3C). MEK/ERK inhibition would then lead to rapid dephosphorylation and proteasomal degradation of this transcriptional repressor (herein called “Inhibitor X”) and thereby derepress *GATA6* transcription. Proteasome inhibition protects Inhibitor X from degradation even during ERK inhibition and therefore counteracts the effect of MEK/ERK inhibition on *GATA6*.

**Figure 4:**
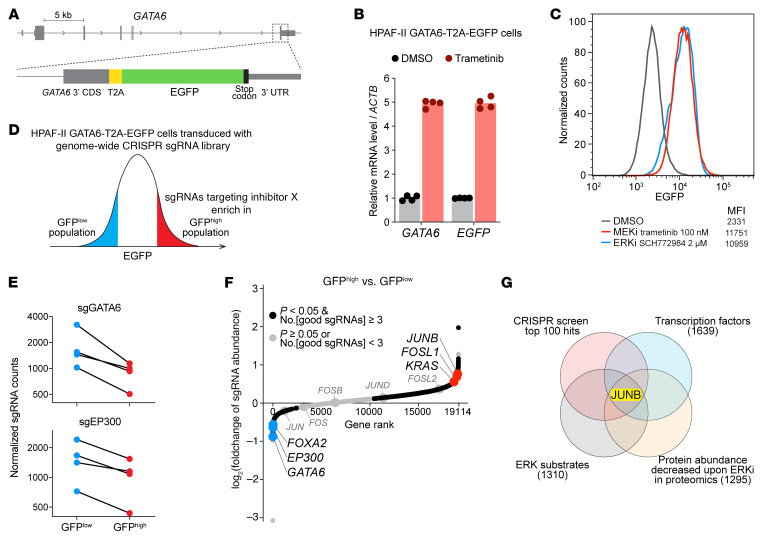
Genome-wide CRISPR screening identifies transcriptional repressor JUNB for *GATA6*. (**A**) Illustration of the knockin of a T2A-EGFP cassette into the *GATA6* locus of HPAF-II cells. CDS, coding sequence. (**B**) *EGFP* transcript faithfully reflected the response of *GATA6* transcription to MEK/ERK inhibition. HPAF-II GATA6-T2A-EGFP cells were treated with DMSO or 100 nM trametinib for 24 h, followed by RT-qPCR analysis. *n* = 2 biological replicates × 2 technical replicates/condition. (**C**) MEK/ERK inhibition upregulated EGFP abundance. HPAF-II GATA6-T2A-EGFP cells were treated with DMSO or the indicated inhibitors for 4 days and analyzed by flow cytometry. The MFI values of EGFP are shown. (**D**) Illustration of the genome-wide CRISPR screening performed in HPAF-II GATA6-T2A-EGFP cells. The top 20% GFP^hi^ and bottom 20% GFP^lo^ cell populations were sorted out for analysis. (**E**) Positive controls validated the CRISPR screen. sgRNAs targeting *GATA6* or *EP300* were depleted in the GFP^hi^ population. For each gene, all 4 individual sgRNAs are shown. (**F**) Genome-wide CRISPR screening identified positive and negative regulators of *GATA6* transcription. 19,114 genes were ranked based on the gene-level, log_2_-transformed fold change of sgRNAs comparing GFP^hi^ versus GFP^lo^ groups. The gene-level values were calculated for each gene using the MAGeCK algorithm combining all sgRNAs targeting that gene. No.[good sgRNAs] is the number of sgRNAs that showed consistent depletion or enrichment of that gene. (**G**) Cross-referencing CRISPR screen results with additional datasets identified JUNB as an ERK-regulated transcriptional repressor of *GATA6*. Genes annotated as transcription factors or ERK substrates were extracted from references 36 and 37. Genes whose protein abundance decreased upon ERK inhibition were extracted from reference 29. Numbers in parentheses refer to the number of genes in each dataset.

To identify the Inhibitor X that links KRAS/ERK signaling to *GATA6* transcription, we performed a genome-wide CRISPR screen looking for genes whose knockout increased *GATA6* transcription. First, we generated a reporter cell line by knocking a T2A-EGFP cassette into the *GATA6* genomic locus of HPAF-II cells so that EGFP RNA and protein would be coexpressed with endogenous GATA6 (Figure 4A). Indeed, the *EGFP* transcript faithfully reflected the change of *GATA6* transcription in response to MEK/ERK inhibition (Figure 4B), and, importantly, flow cytometry analysis showed a substantial increase of EGFP abundance in this reporter cell line after MEK/ERK inhibition (Figure 4C).

We transduced these HPAF-II GATA6-T2A-EGFP cells with the Brunello genome-wide CRISPR lentiviral library (35) that targets 19,114 genes and then used FACS to collect the top 20% GFP^hi^ and the bottom 20% GFP^lo^ cells (Figure 4D). sgRNAs were recovered from these samples and subjected to next-generation sequencing and statistical analysis (Supplemental Table 1). Validating the functionality of our screening, *GATA6* was the topmost hit whose sgRNAs were depleted from the GFP^hi^ cell population (Figure 4, E and F). This is because sgRNAs targeting *GATA6* introduced indels in the *GATA6* coding sequence, causing frameshifts that eliminated expression of the downstream T2A-linked EGFP as well. *EP300* (encoding p300) was another hit reidentified in this screen, as our prior work has shown that loss of p300 decreases *GATA6* transcription (14). The screen also identified *FOXA2*, another lineage-specific transcription factor and classical subtype pancreatic cancer–associated marker gene (31), as a positive regulator of *GATA6* (Figure 4F). While a positive correlation between the expression levels of *FOXA2* and *GATA6* has been reported in pancreatic tumors previously (12), our screen result indicates that FOXA2 could be a direct activator of *GATA6* transcription.

Knockout of negative regulators of *GATA6* transcription, including *KRAS*, led to derepression of *GATA6* transcription; hence, their sgRNAs were enriched in the GFP^hi^ cell population (Figure 4F). According to our hypothesized model (Figure 3C), Inhibitor X should be in this category of genes. Our genome-wide CRISPR screen generated hundreds of hits, so to narrow it down and identify Inhibitor X, we filtered it using additional criteria. Ideally, Inhibitor X should be a transcription factor, it should be a direct/indirect protein target of the ERK, and its protein abundance should decrease upon ERK inhibition. For the filtering, we used a curated list of transcription factors (36), a curated list of ERK targets (37), and a recent study of ERK-regulated proteins (29). After eliminating CRISPR screen hits that were not present in these additional datasets, only a single candidate remained, the transcription factor JUNB (Figure 4G). JUNB is a member of the Jun family of transcriptional regulators; these form heterodimers with other Activating Protein-1 (AP-1) proteins, including Fos family members, to form the AP-1 transcription factor complex (38). Interestingly, the other 2 members of the Jun family, c-JUN and JUND, showed no effect in our CRISPR screen. We also noted that 2 Fos family members, FOSL1 and FOSL2, showed a similar but weaker effect as JUNB in the screen (Figure 4F). We speculate that JUNB can form repressive heterodimers with either FOSL1 or FOSL2.

### JUNB mediates ERK’s suppressive effect on GATA6 transcription.

**Figure 5:**
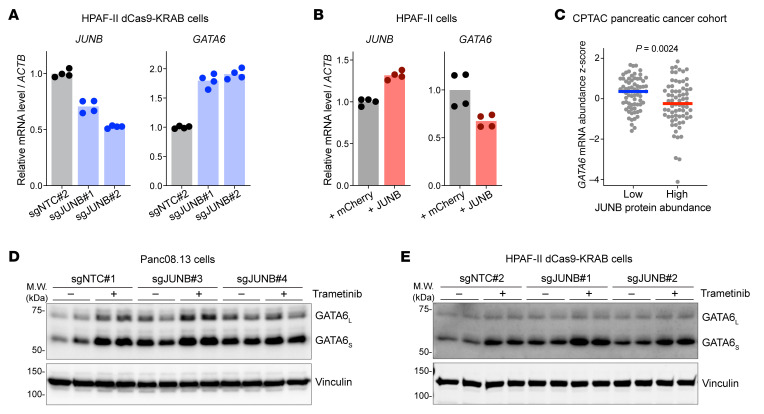
JUNB mediates ERK’s suppressive effect on *GATA6* transcription. (**A**) Knockdown of *JUNB* increased *GATA6* transcript abundance. HPAF-II cells stably expressing dCas9-KRAB fusion protein were transduced with a lentivirus expressing a dox-inducible nontargeting control sgRNA (sgNTC#2) or sgRNAs targeting the *JUNB* promoter (sgJUNB#1 and sgJUNB#2). Cells were treated with dox for 6 days and analyzed by RT-qPCR. *n* = 2 biological replicates × 2 technical replicates/condition. (**B**) Overexpression of JUNB downregulated *GATA6* transcript abundance. HPAF-II cells stably expressing mCherry control or JUNB were analyzed by RT-qPCR. *n* = 2 biological replicates × 2 technical replicates/condition. (**C**) Pancreatic tumors with high JUNB protein abundance showed lower levels of *GATA6* transcript. JUNB protein abundance and *GATA6* mRNA abundance data of human pancreatic tumors in the CPTAC cohort (39) were extracted from the cBioPortal website (85). Each dot represents an individual tumor, and mean values of each group are shown. *n* = 70/group. *P* value of 2-tailed, unpaired *t* test is shown. (**D**) Knockout of *JUNB* in Panc08.13 cells mimicked the effect of MEK/ERK inhibition on GATA6 expression. Panc08.13 cells were transduced with lentivirus expressing Cas9 and a nontargeting control sgRNA (sgNTC#1) or a sgRNA targeting *JUNB* (sgJUNB#3 or sgJUNB#4). After puromycin selection, these genome-edited cell pools were treated with DMSO or 100 nM trametinib for 24 h, followed by Western blot analysis. *n* = 2 biological replicates. (**E**) *JUNB* knockdown in HPAF-II cells mimicked the effect of MEK/ERK inhibition on GATA6 expression. Cells from (**A**) were treated with DMSO or 100 nM trametinib for 24 h, followed by Western blot analysis. *n* = 2 biological replicates.

To independently test if JUNB represses *GATA6* transcription, we used dCas9-KRAB CRISPR interference (CRISPRi) to knock down *JUNB* expression in HPAF-II cells. Indeed, knockdown of *JUNB* using 2 independent sgRNAs markedly increased *GATA6* mRNA abundance in culture (Figure 5A) and in xenografts (Supplemental Figure 5). Conversely, overexpression of *JUNB* reduced *GATA6* mRNA abundance (Figure 5B). We also examined the Clinical Proteomic Tumor Analysis Consortium human pancreatic cancer cohort (39) where tumor samples underwent both transcriptomic and proteomic profiling. Tumors with high JUNB protein abundance showed significantly lower levels of *GATA6* mRNA (Figure 5C), while there was no statistically significant correlation between *GATA6* and Jun family members c-JUN and JUND (Supplemental Figure 6). These results support the model that JUNB suppresses *GATA6* transcription.

We further investigated whether JUNB mediates KRAS/ERK’s suppressive effect on *GATA6*. Pooled Panc08.13 cells with *JUNB* knockout had increased GATA6 protein (most readily appreciated with the GATA6_S_ isoform), largely mimicking the effect of MEK/ERK inhibition (Figure 5D and Supplemental Figure 7). In these *JUNB*-knockout pools, trametinib treatment had only a minimal effect on GATA6 abundance. The small additional increase of GATA6 after trametinib treatment in the *JUNB*-knockout cells could be due to the residual unedited or in-frame edited *JUNB* cells in the pools. A similar result was observed in HPAF-II cells when we partially knocked down *JUNB* with CRISPRi (Figure 5, A and E). Taken together, these results support the model that JUNB, stabilized by oncogenic KRAS/ERK signaling, suppresses the transcription of *GATA6*.

**Figure 6:**
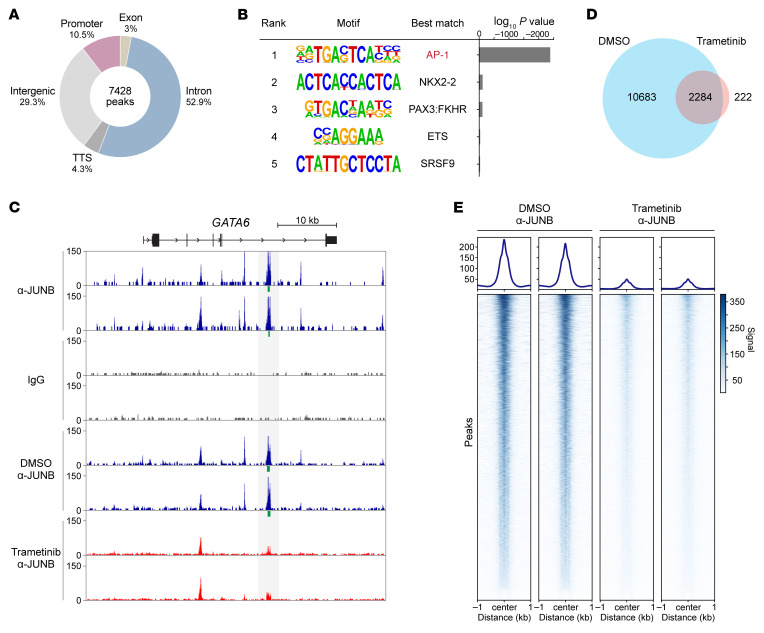
JUNB binds directly to the *GATA6* locus in a MEK/ERK-dependent manner. (**A**) The annotation of genome-wide JUNB binding peaks in untreated HPAF-II cells. Cut&Run-seq was performed in HPAF-II cells using anti-JUNB antibody or IgG control. TTS, transcription termination site. (**B**) De novo motif discovery analysis on JUNB binding peaks shown in (**A**). (**C**) JUNB binds to intron 6 of *GATA6* and responded to trametinib treatment. Cut&Run-seq signals in the *GATA6* locus were visualized in the UCSC Genome Browser. Tracks of anti-JUNB antibody and IgG control for untreated HPAF-II cells and tracks of anti-JUNB antibody for HPAF-II cells treated with DMSO control or 100 nM trametinib for 24 h are shown. The peaks identified by the peak-calling algorithm are shown as green bars, and this region is highlighted in gray in the graph. *n* = 2 biological replicates/condition. (**D** and **E**) Trametinib treatment diminished genome-wide JUNB binding. (**D**) The numbers and overlap of JUNB peaks identified by Cut&Run-seq in DMSO- or trametinib-treated HPAF-II cells. (**E**) Visualization of Cut&Run-seq signals for JUNB peaks identified in DMSO- or trametinib-treated HPAF-II cells. *n* = 2 biological replicates/condition.

JUNB is likely to function as a transcriptional repressor via its sequence-specific DNA binding (40, 41). To determine the genome occupancy and regulation of JUNB in pancreatic cancer, we used anti-JUNB antibody or IgG control to perform a Cut&Run-seq (Cleavage Under Targets and Release Using Nuclease sequencing) assay in HPAF-II cells without or with MEK inhibition with trametinib. In control cells, this assay identified 7,428 JUNB binding peaks, approximately 70% of which are localized in gene loci, especially in intronic regions (Figure 6A). De novo motif discovery analysis on these peaks identified a single highly enriched motif that matches the known AP-1 binding motif (Figure 6B), validating the accuracy of our Cut&Run-seq. Importantly, the peak-calling algorithm identified significant JUNB binding in intron 6 of *GATA6* (Figure 6C). We next examined the effect of MEK/ERK inhibition on JUNB’s genome occupancy. Notably, trametinib treatment diminished genome-wide JUNB binding, decreasing the number of JUNB peaks by approximately 80% (Figure 6, D and E). Importantly, the signal of the JUNB peak in the *GATA6* locus was markedly reduced in the trametinib-treated samples and was no longer recognized as a significant peak (Figure 6C). These results reveal that JUNB binds directly to the *GATA6* locus in a MEK/ERK-dependent manner, suggesting a direct mechanism for *GATA6* repression.

### ERK stabilizes JUNB in a GSK3/FBXW7-independent manner.

**Figure 7:**
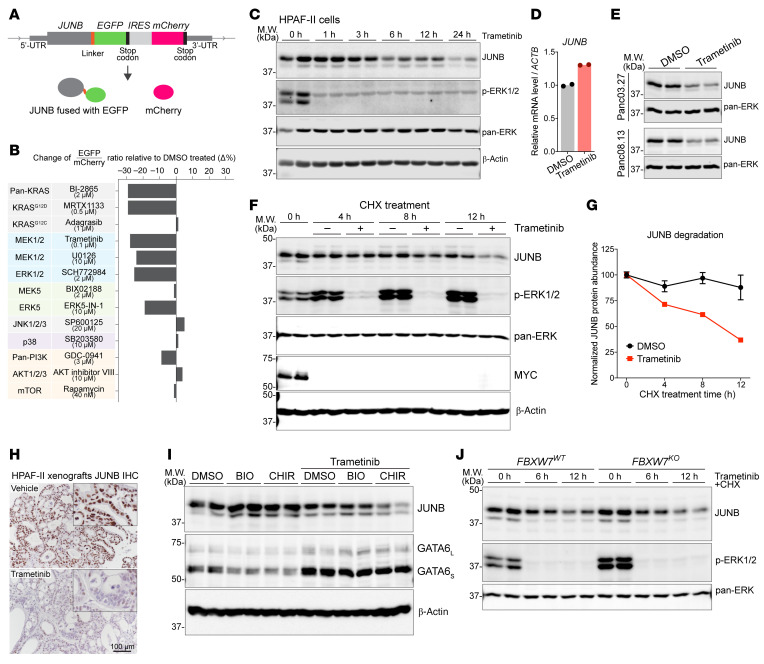
ERK stabilizes JUNB in a GSK3/FBXW7-independent manner. (**A**) Illustration of the knockin of an EGFP-IRES-mCherry cassette into the *JUNB* locus. (**B**) Targeting the KRAS^G12D^-activated MEK/ERK axis decreased JUNB-EGFP fusion protein abundance. HPAF-II JUNB-EGFP-IRES-mCherry cells were treated with small-molecule inhibitors (targets of the inhibitors shown on the left) at the indicated concentrations for 24 h, followed by flow cytometry analysis. (**C**) JUNB protein abundance decreased over time upon MEK/ERK inhibition. HPAF-II cells were treated with 100 nM trametinib for the indicated time. *n* = 2 biological replicates/condition. (**D**) *JUNB* mRNA was not affected by MEK inhibition. HPAF-II cells were treated with DMSO or 100 nM trametinib for 24 h. *n* = 2 technical replicates/condition. (**E**) MEK/ERK inhibition decreased JUNB abundance in Panc03.27 and Panc08.13 cells. Cells were treated with DMSO or 100 nM trametinib for 24 h. *n* = 2 biological replicates/condition. (**F** and **G**) MEK/ERK inhibition promoted JUNB degradation. (**F**) HPAF-II cells were treated with 40 μg/mL CHX with or without 100 nM trametinib for the indicated time. MYC, a short half-life protein, was used as a control for CHX. (**G**) Quantification (mean ± SD) of JUNB bands. *n* = 2 biological replicates/condition. (**H**) MEK/ERK inhibition decreased JUNB abundance in vivo. HPAF-II tumors from the study shown in Figure 2D were stained for JUNB. Scale bar: 100 μm. (**I**) MEK/ERK inhibition–caused JUNB degradation was independent of GSK3 activity. HPAF-II cells were treated with DMSO or a GSK3 inhibitor (5 μM BIO or 5 μM CHIR99021) with or without 100 nM trametinib for 24 h. *n* = 2 biological replicates/condition. (**J**) MEK/ERK inhibition–caused JUNB degradation was independent of FBXW7. HPAF-II cells with WT or homozygous knockout of *FBXW7* were treated with 40 μg/mL CHX and 100 nM trametinib for the indicated time. *n* = 2 biological replicates/condition.

We next investigated how KRAS/ERK signaling regulates JUNB protein turnover in pancreatic cancers. We knocked an EGFP-IRES-mCherry cassette into the *JUNB* locus in HPAF-II cells so that the endogenous JUNB protein was expressed as a fusion at the C-terminus with EGFP, with coexpression of mCherry protein (Figure 7A). The change in ratio of EGFP to mCherry then reflected posttranscriptional regulation of JUNB protein abundance. With this reporter system, we examined a panel of 13 inhibitors targeting key nodes of the KRAS/MEK/ERK and related signaling pathways. Importantly, only inhibitors targeting the KRAS^G12D^-activated MEK/ERK axis led to a consistent and robust decrease of JUNB abundance (Figure 7B), further supporting the model that oncogenic KRAS/ERK signaling stabilizes JUNB protein.

The time course of JUNB protein degradation upon MEK/ERK inhibition was examined. A decrease in JUNB abundance was detected as early as 3 h after treatment (Figure 7C), which inversely correlated with the increase in *GATA6* transcript (Figure 2B). The decrease of JUNB protein was not due to changes in *JUNB* transcription (Figure 7D). Similar results were observed in 2 additional *KRAS*-mutant pancreatic cancer cell lines (Figure 7E). In a cycloheximide (CHX) chase assay, JUNB protein was stable when ERK signaling was active but was degraded upon MEK/ERK inhibition (Figure 7, F and G). This also suggests that the decrease of JUNB abundance by MEK/ERK inhibition was due to destabilization of JUNB protein. Moreover, MEK/ERK inhibition reduced JUNB abundance in HPAF-II xenografts (Figure 7H), confirming that this regulation also occurs in vivo.

GSK3 and FBXW7 have been reported to promote JUNB degradation. In that model, GSK3 phosphorylates a degron site in JUNB that is then recognized by the FBXW7 SCF E3 ligase complex, leading to ubiquitylation and proteasomal degradation (42). Indeed, treatment of HPAF-II cells with GSK3 inhibitors BIO and CHIR99021 alone led to a modest increase in JUNB and equally modest decrease in GATA6 protein abundance, supporting the existence of basal GSK3-dependent JUNB degradation (Figure 7I). We next tested if MEK/ERK inhibition promoted JUNB degradation through the GSK3/FBXW7 pathway. GSK3 inhibition could not prevent JUNB degradation caused by MEK/ERK inhibition (Figure 7I). Similarly, *FBXW7* knockout showed no effect on JUNB degradation upon MEK/ERK inhibition (Figure 7J and Supplemental Figure 8). These results indicate that ERK inhibition drives JUNB degradation independent of GSK3 and FBXW7.

### ERK inhibition promotes ubiquitin-independent proteasomal degradation of JUNB.

Notably, 2 paralogs of JUNB, c-Fos and Fra-1, have been reported to undergo ubiquitin-independent proteasomal degradation (43, 44). As the ERK-regulated JUNB turnover was independent of GSK3/FBXW7, we examined its dependency on the ubiquitin-proteasome pathway. To assess this, we treated cells with (a) proteasome inhibitor bortezomib or MG132 that blocks proteasomal degradation, (b) the ubiquitin-activating E1 enzyme inhibitor MLN7243 that blocks ubiquitylation (45), or (c) the NEDD8-activating E1 enzyme inhibitor MLN4924 that blocks neddylation (46). Notably, neddylation, a ubiquitylation-like process, can directly regulate the function and/or turnover of certain substrates, and JUNB was reported to be a neddylation substrate (47). Neddylation is also necessary for the activity of cullin RING ligases, the largest family of E3 ubiquitin ligases, including the above-mentioned FBXW7 SCF ligase complex (48).

**Figure 8:**
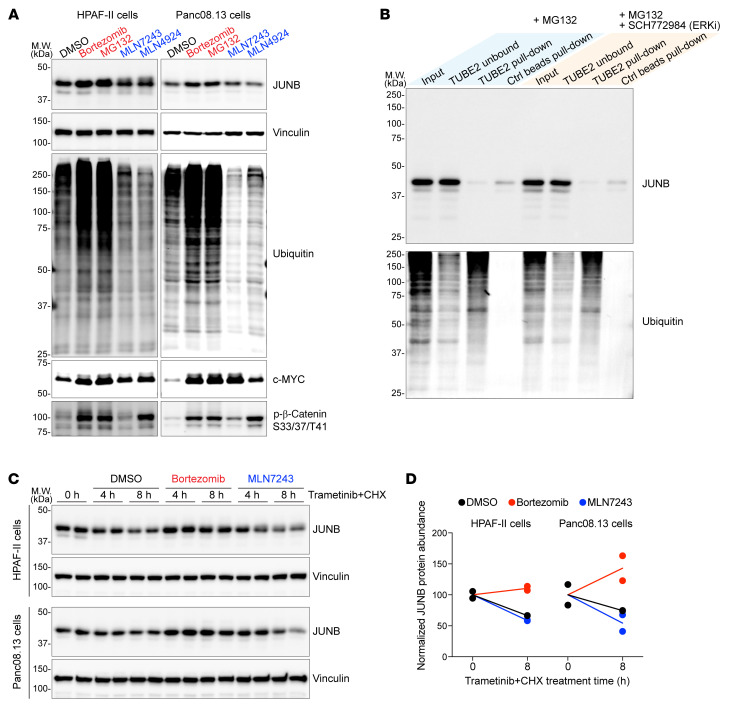
ERK inhibition promotes ubiquitin-independent proteasomal degradation of JUNB. (**A**) Inhibition of the proteasome, rather than ubiquitylation or neddylation, stabilized JUNB. HPAF-II and Panc08.13 cells were treated with DMSO or inhibitors of the proteasome (1 μM bortezomib or 10 μM MG132), ubiquitylation (1 μM MLN7243), or neddylation (1 μM MLN4924) for 7 h, followed by Western blot analysis. (**B**) JUNB was not ubiquitylated during degradation. HPAF-II cells were treated with 10 μM MG132 with or without 2 μM ERK inhibitor SCH772984 for 6 h. Cell lysates were pulled down using TUBE2-conjugated beads that captured polyubiquitylated proteins. Control (Ctrl) beads refers to beads without TUBE2 conjugation. (**C** and **D**) Inhibition of proteasome, rather than ubiquitylation, blocked JUNB degradation induced by MEK/ERK inhibition. (**C**) HPAF-II and Panc08.13 cells were treated with 40 μg/mL CHX and 100 nM trametinib for the indicated time, in the presence of DMSO or inhibitors of the proteasome (1 μM bortezomib) or ubiquitination (1 μM MLN7243), followed by Western blot analysis. (**D**) Quantification of JUNB bands. *n* = 2 biological replicates/condition.

As expected, proteasome inhibition led to global accumulation of ubiquitylated proteins, while ubiquitylation or neddylation inhibition showed the opposite effect (Figure 8A). Additional positive controls included MYC, a ubiquitin-dependent proteasomal substrate, and β-catenin, where phosphorylation on residues Ser33/37 promotes ubiquitin-independent but neddylation-dependent proteasomal degradation of β-catenin (49). Interestingly, only proteasome inhibition (which could only be applied for 7 h, ~1 JUNB half-life, due to cytotoxicity of the doses used) led to JUNB accumulation, while inhibiting either ubiquitylation or neddylation had no effect on JUNB abundance (Figure 8A). This observation implies ubiquitin-independent proteasomal degradation of JUNB. Moreover, pulling down ubiquitylated proteins from lysate of proteasome inhibitor–treated cells could not enrich JUNB regardless of ERK activity (Figure 8B), indicating that JUNB was not ubiquitylated during degradation. Finally, proteasome inhibition, rather than ubiquitylation inhibition, blocked JUNB degradation induced by MEK/ERK inhibition (Figure 8, C and D). Taken together, these results show that ERK inhibition promotes proteasomal degradation of JUNB in a ubiquitin-independent manner.

### Upregulating GATA6 enhances sensitivity of pancreatic cancer cells to oxaliplatin.

**Figure 9:**
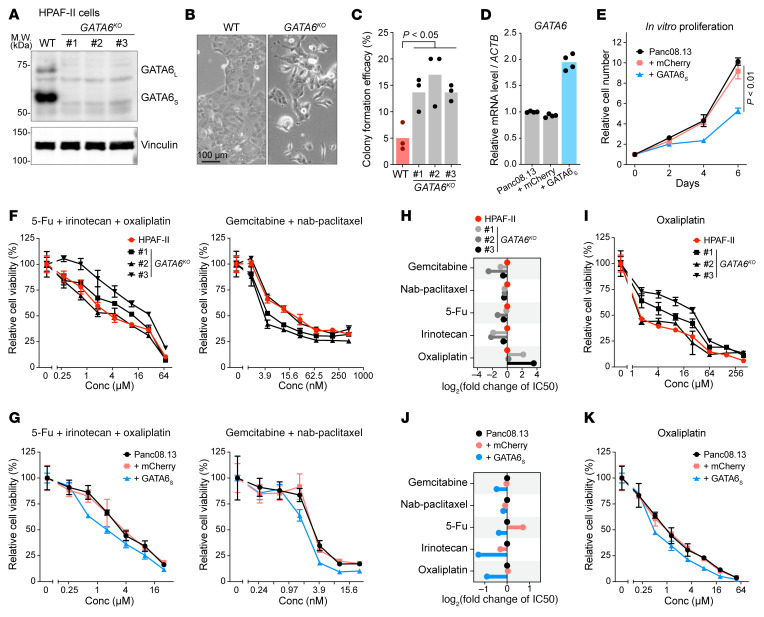
Upregulating GATA6 enhances chemosensitivity of pancreatic cancer cells. (**A**–**C**) *GATA6* knockout led to EMT and increased stemness in HPAF-II cells. (**A**) Western blotting confirmed the knockout of *GATA6*. Numbers 1–3 are 3 single-cell–derived clonal *GATA6^KO^* lines. (**B**) Representative images showing EMT morphology of *GATA6^KO^* cells. Scale bar: 100 μm. (**C**) *GATA6^KO^* lines showed increased colony formation capacity when single cells were seeded in 96-well plates. *n* = 3 biological replicates/condition. The *P* value of Dunnett’s multiple-comparison test is shown. (**D** and **E**) Overexpressing *GATA6* slowed the proliferation of Panc08.13 cells. (**D**) Panc08.13 cells were transduced with lentivirus expressing GATA6_S_ or mCherry as a control. *n* = 2 biological replicates × 2 technical replicates/condition. (**E**) Cells were seeded in 96-well plates, and cell proliferation was monitored with the CellTiter-Glo assay. *n* = 3 biological replicates/condition. *P* values of 2-tailed, unpaired *t* test are shown. (**F** and **G**) *GATA6* affected sensitivity to standard-of-care chemotherapy combinations in HPAF-II (**F**) and Panc08.13 (**G**) cells. The concentration (Conc) shown on the *x* axes of these plots refers to the concentration of 5-FU or gemcitabine in the drug combinations. *n* = 2–3 biological replicates/condition. (**H** and **I**) *GATA6* knockout conferred resistance to oxaliplatin in HPAF-II cells. (**H**) Responses to individual chemotherapy agents were examined for *GATA6* WT and knockout HPAF-II cells. Changes in IC_50_ values relative to WT HPAF-II cells are shown. (**I**) The dose–response curve to oxaliplatin is shown. *n* = 2–3 biological replicates/condition. (**J** and **K**) Overexpressing *GATA6* sensitized Panc08.13 cells to irinotecan and oxaliplatin. (**J**) Responses to individual chemotherapy agents were examined for parental Panc08.13 as well as GATA6_S_- or mCherry-overexpressing cells. Changes in IC_50_ values relative to WT Panc08.13 cells are shown. (**I**) The dose–response curve to oxaliplatin is shown. *n* = 2–3 biological replicates/condition. Data are shown as the mean ± SD.

High *GATA6* expression leads to pancreatic cancer differentiation and correlates with superior therapy response and longer survival for pancreatic cancer patients (5, 7, 12, 50). To directly test if GATA6 expression levels determine chemosensitivity, we knocked out or overexpressed *GATA6* in 2 pancreatic cancer cell lines. Consistent with the prodifferentiation role of GATA6 (12, 14), *GATA6* knockout led to epithelial-mesenchymal transition (EMT) features in HPAF-II cells and increased the colony formation capacity of single cancer cells (Figure 9, A–C). Conversely, moderate overexpression of *GATA6* significantly slowed down the proliferation of Panc08.13 cells (Figure 9, D and E). These observations further validate the tumor suppressor roles of GATA6 in pancreatic cancers. At the molecular level, knockout of *GATA6* in HPAF-II cells led to consistent downregulation of all the tested classical subtype genes. We did not observe upregulation of the tested basal subtype genes (Supplemental Figures 9 and 10). This is consistent with our previous study where we found that downregulating *GATA6* by either naturally existing p300 mutations or by experimental p300 knockout downregulated classical subtype genes but did not upregulate basal subtype genes (14). We speculate that this might represent an initial stage in the transition from classical to basal subtype.

Currently there are 2 main standard-of-care chemotherapy regimens for first-line treatment of pancreatic cancers (3): (a) modified FOLFIRINOX that combines leucovorin, 5-fluorouracil (5-FU), irinotecan, and oxaliplatin; and (b) gemcitabine with nab-paclitaxel. The GATA6-high classical subtype is notably more response to therapy. We examined how manipulation of GATA6 affected the sensitivity to these standard-of-care regimens. Notably, compared with the parental HPAF-II cells, 2 out of 3 *GATA6*-knockout lines became less sensitive to the combination of 5-FU, irinotecan, and oxaliplatin, while this effect was not evident for the gemcitabine/nab-paclitaxel combination (Figure 9F and Supplemental Table 2). Conversely, overexpressing *GATA6* in Panc08.13 cells increased their sensitivity to both drug combination regimens (Figure 9G and Supplemental Table 2).

We further assayed the response to individual chemotherapeutic agents. HPAF-II cells with *GATA6* knockout showed specific resistance to oxaliplatin (Figure 9, H and I, and Supplemental Table 2); these cells tended to be more sensitive to gemcitabine and irinotecan, while their sensitivity to nab-paclitaxel and 5-FU was not affected. As these *GATA6*-knockout cells underwent single-clone isolation that might affect their behavior, we also performed the drug sensitivity assays in cell pools transduced with control or *GATA6*-targeting sgRNAs (Supplemental Figures 9 and 11, and Supplemental Table 2). Consistent with results obtained with the clonal *GATA6*-knockout lines, the *GATA6*-knockout cell pools tended to be less sensitive to oxaliplatin but more sensitive to gemcitabine and irinotecan, albeit the effects were weaker than in the *GATA6* complete knockout clonal lines. In Panc08.13 cells, *GATA6* overexpression showed a general chemo-sensitizing trend, and this effect was more evident for irinotecan and oxaliplatin (Figure 9, J and K, and Supplemental Table 2), both from the FOLFIRINOX regimen. In vivo, while Panc08.13 xenografts were quite resistant to oxaliplatin, the GATA6-overexpressed tumors tended to be more sensitive to oxaliplatin; the ratio of tumor growth inhibition by oxaliplatin was 12.5% in the mCherry control group versus 20.5% in GATA6-overexpressing group (Supplemental Figure 12). Compared with the control group (mCherry + vehicle treatment), GATA6 overexpression plus oxaliplatin significantly reduced tumor growth, while either one alone was less effective. Taken together, increased GATA6 expression could increase chemosensitivity, especially for oxaliplatin, in pancreatic cancer models.

### KRAS/ERK inhibition synergizes with oxaliplatin in pancreatic cancer models.

**Figure 10:**
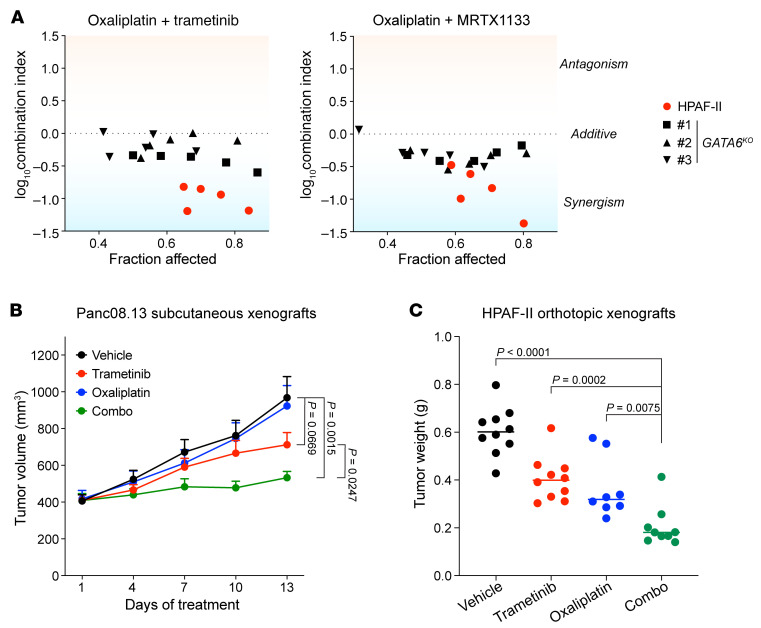
KRAS/ERK inhibition synergizes with oxaliplatin in pancreatic cancer models. (**A**) *GATA6*-dependent drug synergy between oxaliplatin and KRAS/MEK/ERK inhibitors. Drug combination effects between oxaliplatin and the MEK inhibitor trametinib or the KRAS^G12D^ inhibitor MRTX1133 were examined for *GATA6* WT or knockout HPAF-II cells following the Chou-Talalay protocol. The fraction affected refers to growth inhibitory effects of the drug combinations (0, no inhibition; 1, 100% inhibition), and the corresponding log_10_-transformed Combination Index values are shown. The smaller the Combination Index value, the stronger the drug synergism. (**B** and **C**) Trametinib and oxaliplatin combination treatment potently inhibited pancreatic tumor growth in xenograft models. (**B**) Mice bearing Panc08.13 subcutaneous xenografts were treated with vehicle, trametinib (1.5 mg/kg per dose, twice weekly), oxaliplatin (5 mg/kg per dose, once weekly), or the combination of trametinib and oxaliplatin (combo) for 2 weeks. *n* = 9–12 tumors per arm. Data are shown as the mean ± SEM. (**C**) Mice bearing HPAF-II orthotopic xenografts were treated with vehicle, trametinib (0.5 mg/kg per dose × 10 doses administered over 4 weeks), oxaliplatin (5 mg/kg per dose, twice weekly), or the combination of trametinib and oxaliplatin (combo) for 4 weeks. *n* = 8–10 tumors per arm. *P* values of 2-tailed, unpaired *t* test are shown.

Oncogenic KRAS/ERK signaling inhibits *GATA6* transcription, providing a clinically relevant way to upregulate GATA6. Given the potential of chemosensitization by upregulating GATA6, small-molecule inhibitors of the KRAS/MEK/ERK axis might synergize with chemotherapy agents for treating pancreatic cancers. Indeed, in HPAF-II cells, we observed strong synergy between oxaliplatin and both the MEK inhibitor trametinib and the KRAS^G12D^ inhibitor MRTX1133 (Figure 10A). Notably, the drug synergy was substantially diminished in *GATA6*-knockout cells (Figure 10A and Supplemental Figure 13), indicating that the synergy between oxaliplatin and KRAS/MEK/ERK inhibitors was largely mediated by the upregulation of GATA6. We next examined the drug combination strategy in vivo. In both Panc08.13 subcutaneous xenografts and HPAF-II orthotopic xenografts, trametinib and oxaliplatin combination treatment showed synergistic or at least additive effects on tumor growth inhibition (Figure 10, B and C). Thus, combining KRAS/ERK inhibitors with standard-of-care chemotherapies could be a promising therapeutic strategy for treating pancreatic cancers.

## Discussion

Several studies have established a *GATA6*-centered regulatory network that determines pancreatic cancer states, but why *GATA6* expression levels vary across tumors and disease stages remains elusive. Our current study revealed a robust regulatory axis where oncogenic KRAS/ERK signaling stabilizes the transcriptional repressor JUNB to inhibit *GATA6* expression. These findings link the major oncogenic driver of pancreatic cancer, hyperactivated KRAS signaling, to *GATA6*. *KRAS* hotspot mutations are prevalent in pancreatic cancers, but the downstream signaling outputs are not uniform for all patients. This could result from variations in *KRAS* mutant alleles and copy numbers, e.g., due to the frequent amplification of a mutant *KRAS* allele in late-stage pancreatic tumors (15, 17, 51). The involvement of upstream growth factors and their receptors can further modulate KRAS activity (52, 53). The combination of these factors contributes to variable levels of KRAS/ERK activation and, therefore, to the heterogeneity of *GATA6* expression in pancreatic cancers.

Oncogenic activation of KRAS signaling is fundamental to the initiation and maintenance of pancreatic cancer, and its pro-proliferative and anti-apoptotic functions and mechanisms have been extensively studied (54, 55). In contrast, while phenotypically the antidifferentiation role of oncogenic KRAS has also been recognized during the early stages of pancreatic tumorigenesis (56, 57), its molecular mechanism remains elusive. Given our finding that KRAS/ERK signaling suppresses *GATA6* expression and GATA6 is a master regulator of differentiation in pancreatic lineage, this previously unrecognized KRAS/ERK/JUNB/GATA6 axis could be a key mechanism of the dedifferentiation caused by oncogenic KRAS. Notably, recent studies observed that treating pancreatic tumors with the KRAS^G12D^ inhibitor MRTX1133 led to a rapid enrichment of cancer cells with the classical state (18, 19). We speculate this was because KRAS inhibition upregulated GATA6, which led to cancer subtype switching from basal-like to classical state. Interestingly, KRAS inhibition in *KRAS*-mutant lung cancer similarly led to a switch of the cancer cell states (58, 59), suggesting a paradigm of the oncogenic KRAS signaling in promoting cancer dedifferentiation. However, whether this is also mediated by the KRAS/JUNB/GATA6 differentiation axis or through another molecular mechanism remains to be explored.

Notably, acute knockout of *GATA6* in HPAF-II cells downregulated the expression of classical subtype genes but did not upregulate the tested basal subtype genes. We speculate that repression of *GATA6* might represent an initial stage in the transition from classical to basal subtype. Supporting this, GATA6 has been shown to be the primary gatekeeper of the classical phenotype, while additional events such as loss of HNF1A/4A and gain of ΔNp63 are needed for a complete transition to the basal subtype (13). This might also partially explain the stronger drug resistance phenotype observed in some clonal *GATA6*-knockout lines versus the acute *GATA6-*knockout cell pools, as these clones might have gained more basal-like features during the single-clone establishment process. Nevertheless, partial loss of classical subtype features upon acute GATA6 reduction is sufficient to lead to a phenotypic switch, including tumor histology and drug sensitivity, as shown in our previous (14) and current study. The loss of GATA6 is permissive for subsequent phenotypic evolution to occur, but this likely takes time and leads to the clonal variability.

Hyperactive ERK suppresses *GATA6* by stabilizing the transcriptional repressor JUNB, while ERK inhibition leads to ubiquitin-independent proteasomal degradation of JUNB. Notably, there are a variety of proteins that can be degraded by the proteasome in the absence of ubiquitylation, and under certain circumstances different pools of the same protein can be targeted to the proteasome by either ubiquitin-dependent or -independent mechanisms (60, 61). Our results show that JUNB is another member of this class of proteasomal substrates. Mechanisms of ubiquitin-independent proteasomal degradation can be diverse (60, 61). In our case, the degradation of JUNB is antagonized by active ERK. Several mass spectrometry studies have proposed JUNB to be a direct substrate of ERK (37, 62, 63). We speculate that the direct phosphorylation by ERK might be important in decreasing JUNB turnover.

Pharmacologically targeting the KRAS/MEK/ERK axis substantially upregulated *GATA6*, which has important clinical implications. Boosting GATA6 expression may improve patient outcomes. Several clinical studies in pancreatic cancers have linked high GATA6 expression and the classical subtype to chemosensitivity (5, 7, 12, 50), and the COMPASS trial found that high GATA6 brought greater clinical benefits for patients treated with modified FOLFIRINOX but less so for patients receiving gemcitabine/nab-paclitaxel treatment (5, 64). Interestingly, this is consistent with our in vitro observation that GATA6 affected sensitivities to individual agents of FOLFIRINOX, especially oxaliplatin, more than that of gemcitabine/nab-paclitaxel, while the mechanism remains to be determined. In preclinical models, combining KRAS/ERK inhibitors with oxaliplatin showed GATA6-dependent synergistic effects. This suggests a mechanism-based therapeutic strategy for enhanced therapy of pancreatic cancer, and as such, its efficacy and safety warrant further investigation.

## Methods

### Sex as a biological variable.

Both male and female mice were used in this study. Sex was not considered as a biological variable.

### Cell culture.

HPAF-II, Panc03.27, and Panc08.13 cells were from the Duke Cell Culture Facility. HPAF-II cells were cultured in Eagle’s Minimum Essential Medium with 10% FBS. Panc03.27 and Panc08.13 cells were cultured in RPMI 1640 medium with 15% FBS and 4 μg/mL human recombinant insulin (Gibco, 12585014). All culture media contained 1 mM sodium pyruvate, 2 mM l-glutamine, and 1% (v/v) penicillin/streptomycin (Gibco, 15140122) unless otherwise stated. Cells were cultured in a humidified incubator with 5% CO_2_.

For JUNB overexpression, the *JUNB* sequence was PCR amplified from the genomic DNA of HPAF-II cells using primers described in Supplemental Table 3 and cloned into the BamHI and XhoI double-digested pLenti6/V5-D-TOPO vector. The lentiviral GATA6_S_-expressing plasmid (pLenti6/V5-D-TOPO vector) generated in our previous study (14) was used for GATA6 stable overexpression. The pLenti6-V5-mCherry plasmid (Addgene, 128062, a gift from Oskar Laur, Emory University, Atlanta, Georgia) was used as a control. Lentiviruses were produced as described previously (14). Cells were transduced with lentivirus in the presence of 8 μg/mL polybrene. Cells were selected with 20 μg/mL blasticidin for 1 week and maintained in blasticidin-free media.

### CRISPR genome/epigenome editing.

sgRNAs described in Supplemental Table 2 were cloned into lentiCRISPR v2 (Addgene, 52961, a gift from Feng Zhang, Broad Institute, Cambridge, Massachusetts) (65) or the dox-inducible sgRNA expression vector FgH1tUTG (Addgene, 70183, a gift from Marco Herold, Walter and Eliza Hall Institute of Medical Research, Parkville, Victoria, Australia) (66) following the protocol described previously (65, 66). To knock down *JUNB* in HPAF-II cells with CRISPRi, cells constitutively expressing dCas9-KRAB fusion protein from our previous study (67) were transduced with FgH1tUTG lentivirus with a sgRNA targeting the *JUNB* promoter region or a nontargeting control sequence. These cells were treated with 1 μg/mL dox for 6 days and subjected to RNA extraction and RT-qPCR or Western blotting. In Panc08.13 cells, *JUNB* was knocked out following previously established protocols (14), and the genome-edited cell pools were used for biochemical assays. *FBXW7* was knocked out in HPAF-II cells as previously described (67), and single-cell–derived clones were established. *GATA6* was knocked out in HPAF-II cells either by electroporating the lentiCRISPR v2 plasmid with sgRNAs targeting *GATA6* into these cells followed by single-cell–derived clone establishment or by transducing cells with lentiCRISPR v2 lentivirus where the genome-edited cell pools were used for subsequent assays. For genotyping, genomic DNA was extracted followed by PCR amplification with primers flanking the expected cutting sites. PCR amplicons were gel purified and subject to Sanger sequencing. The indels were visualized using TIDE (68) for genome-edited cell pools.

Fluorescence reporter cassettes were knocked into the *GATA6* or *JUNB* loci in HPAF-II cells using a CRISPR-based strategy (69). sgRNA sequences targeting the *GATA6* or *JUNB* loci, described in Supplemental Table 3, were cloned into lentiCRISPR v2 vector. Knockin repair templates with the structure, sgRNA sequence + 150 bp homology arm + fluorescence reporter cassette sequence to knock in + 150 bp homology arm + sgRNA sequence, were synthesized by Gene Universal Inc. and cloned in the pUC57 vector. Detailed information on the repair templates is provided in Supplemental Figure 14. The sgRNA and Cas9 plasmid (lentiCRISPR v2) and repair template plasmid (pUC57) were coelectroporated into HPAF-II cells using the Thermo Fisher Scientific Neon Electroporation system. The electroporated cells were cultured in antibiotic-free media for 2 days followed by selection with 2 μg/mL puromycin for 2 days. Single-cell–derived subclones were established and subjected to genotyping and Sanger sequencing.

### Genome-wide CRISPR screening.

HPAF-II GATA6-T2A-EGFP cells were transduced with the Brunello human CRISPR knockout pooled library (35) in LentiCRISPR v2 backbone at MOI = 0.3. 5 × 10^7^ HPAF-II cells were transduced with this library of 76,441 sgRNAs to ensure library coverage of approximately 200 cells/sgRNA given the MOI. Two days after transduction, cells were selected with 2 μg/mL puromycin for 3 days and cultured in puromycin-free media for 2 days. Cells were then trypsinized into single cells, suspended in 3% (v/v) FBS in PBS with 0.5 mM EDTA, and subjected to FACS. The top 20% EGFP^hi^ and bottom 20% EGFP^lo^ cell populations, each with ~6 × 10^6^ cells (library coverage of ~80 cells/sgRNA) were sorted out. Genomic DNA from these samples were extracted using the Qiagen Gentra Puregene protocol followed by PCR amplification of the sgRNA cassette using primers described in Supplemental Table 4. Gel-purified PCR amplicons were quantified with the KAPA Library Quantification Kit (KK4828) and sequenced using the HiSeq PE150 (Illumina).

The sequencing fastq files were processed using the processAmplicons package (70) from the edgeR library to count the read number of each sgRNA in each sample. No mismatch of sgRNA sequence was allowed. MAGeCK (71) was used to compare the EGFP^hi^ versus the EGFP^lo^ populations and identify the depleted or enriched sgRNAs and the corresponding genes.

### Cut&Run-seq.

The Cut&Run assay for JUNB was performed in HPAF-II cells using the CUT&RUN Assay Kit (Cell Signaling Technology, 86652) following the manufacturer’s protocol. 200,000 untreated cells or cells treated with DMSO control or 100 nM trametinib for 24 h were used for each reaction. Anti-JUNB antibody (Cell Signaling Technology, 3753) and rabbit IgG isotype from the CUT&RUN Assay Kit were used. DNA was purified by a Zymo Research Oligo Clean & Concentrator column. The libraries were constructed using the NEBNext Ultra II library preparation kit and the NEBNext Multiplex Oligos for Illumina kit, followed by sequencing on the NovaSeq PE150 platform (Illumina).

Adapter sequences were trimmed with Cutadapt v3.5 (72); paired-end reads were discarded if either mate was < 15 nt after trimming. Trimmed reads were aligned to the human reference genome (GRCh38/hg38; Ensembl release 110 annotation) with Bowtie2 v2.5.4 (73) using default settings, and only alignments with mapping quality ≥ 20 were retained for downstream analyses. For each biological replicate, JUNB peaks were called against the matched IgG control using MACS2 v2.2.7.1 (74) (FDR ≤ 0.01). Reproducible peaks were defined as regions with reciprocal overlaps of ≥20% peak length between replicates using bedtools v2.27.0 (75) (intersect -r -f 0.20). Reproducible peaks were used for peak annotation and motif enrichment with HOMER suite v4.11 (76), using the exact peak widths (-size given).

Genome coverage was computed using bedtools genomecov. Samples were normalized to their read counts within the predefined Cut&Run greenlist regions (77). To visualize global differences in JUNB peaks between DMSO and trametinib treatment groups, signals around peak regions were summarized using deepTools (78) computeMatrix at ±1,000 bp windows centered on peak centers, and the resulting matrix was visualized with plotHeatmap.

### In vivo studies.

In vivo experiments were performed in NOD scid gamma (NSG) mice (79) purchased from The Jackson Laboratory (JAX:005557) and bred and housed in specific pathogen-free conditions. Cells were harvested from cell culture, washed with cold PBS, and resuspended in ice-cold 50% Matrigel (Corning, 354248) in PBS. For subcutaneous implantation, 5 × 10^6^ cells in 200 μL 50% Matrigel were injected into the flanks of NSG mice. Tumor dimensions were measured with a caliper, and tumor volumes were calculated as 0.5 × length × (width)^2^. For orthotopic implantation, 1 × 10^6^ cells in 60 μL 50% Matrigel were injected into the pancreas of NSG mice. After tumor establishment, tumor-bearing mice were randomized and received the indicated treatments. For drug administration, trametinib was formulated in 50% (v/v) PEG400 in water and administered by oral gavage at a dosing volume of 5 μL/g mouse body weight each time. Oxaliplatin was formulated in PBS and administered by i.p. injection at a dosing volume of 5 μL/g mouse body weight per injection. Control groups received corresponding vehicle treatments. For the in vivo CRISPRi experiment, after tumor establishment, mice were fed ad libitum with standard diet containing 600 mg dox/kg (Specialty Feeds, SF08-026) to induce the sgRNA expression. Tumors were harvested and snap-frozen in liquid nitrogen followed by storage at –80°C or immediately fixed in 10% neutral buffered formalin for 2 days at room temperature followed by paraffin embedding.

### Immunofluorescence and immunohistochemical staining.

HPAF-II cells cultured on glass coverslips were treated with the drug as indicated. Cells were washed with PBS and then fixed with 1% PFA at room temperature for 15 min. After 3 washes with PBS for 5 min each, cells were permeabilized by incubation in 0.1% (v/v) Triton X-100 in PBS at room temperature for 15 min, followed by 2 washes with PBS. For blocking, cells were incubated in 2% BSA (g/mL) in PBS at room temperature for 30 min. Cells were then incubated with primary antibodies diluted in 2% BSA in PBS at 4°C overnight, followed by 3 washes with PBS. Cells were subsequently incubated with secondary antibodies diluted in 2% BSA in PBS at room temperature for 1 h, followed by 3 washes with PBS. Cells were then mounted with medium containing DAPI and imaged with a Zeiss LSM710 confocal microscope. Formalin-fixed, paraffin-embedded tissue sections were deparaffinized and rehydrated. Antigen retrieval was performed in 10 mM sodium citrate buffer (pH 6.0). IHC staining was performed following standard methods. The stained sections were dehydrated and mounted using DPX. Bright-field images were taken with a Nikon ECLIPSE Ni-E microscope. Primary and secondary antibodies are described in Supplemental Table 5.

### Western blotting.

Cells were lysed in 4% (g/mL) SDS in water and homogenized by sonication or by passing the lysate through an 18-gauge needle attached to a 1 mL syringe multiple times until a homogeneous lysate was achieved. The concentration of total protein in the lysate was quantified by the bicinchoninic acid (BCA) assay. Equal amounts of total protein of each sample were resolved by 10% SDS-PAGE followed by wet transfer to PVDF membranes. Western blotting was performed following standard methods. Primary and secondary antibodies are described in Supplemental Table 5.

### RNA extraction and qPCR.

Total RNA was extracted using the Qiagen RNeasy kit and reversely transcribed into cDNA using the Bio-Rad iScript cDNA synthesis kit. Real-time qPCR was performed using the Bio-Rad SsoAdvanced Universal SYBR Green Supermix reagent on Bio-Rad CFX96 or CFX384 Touch or Thermo Fisher Scientific QuantStudio 5 real-time PCR machines. Primer sequences are described in Supplemental Table 4.

### Pull-down of ubiquitylated proteins.

HPAF-II cells seeded in 10 cm dishes were treated with the indicated inhibitors. After treatment, cells were washed with ice-cold PBS and lysed in 500 μL buffer containing 50 mM Tris-HCl (pH 7.5), 150 mM NaCl, 1 mM EDTA, 1% NP-40, 10% glycerol, 1× cOmplete Protease Inhibitor Cocktail (Roche, 11873580001), and 1× PhosSTOP (Roche, 4906845001). Cell lysates were homogenized by passing the lysate through an 18-gauge needle attached to a 1 mL syringe approximately 10 times and then clarified by centrifugation at 14,000*g* for 10 min at 4°C. The supernatants were collected, measured for protein concentrations by BCA, and used for the pull-down assay. The pull-down experiment was performed using TUBE2-conjugated magnetic beads (LifeSensors, UM402M) following the manufacturer’s protocol. The Control Magnetic Beads (LifeSensors, UM400M) were used as negative control. 30 μL magnetic beads was used for 300 μg protein in 150 μL reaction volume.

### Cell viability assay.

Cells were seeded in 96-well white-wall clear-bottom plates (Greiner, 655098) with 1,000 cells in 100 μL culture media per well. Two days after seeding, 100 μL fresh media containing drugs was added to each well to reach the indicated drug concentrations in each experiment. For the treatment with drug combination, drugs were combined with a constant ratio according to the IC_50_ of individual drugs for HPAF-II or Panc08.13 cells (IC_50_ value of drug 1 + IC_50_ value of drug 2 + …). In detail, 5-FU, irinotecan, and oxaliplatin were combined (μM:μM:μM) with a 7:3:1 ratio for HPAF-II cells and a 10:2.5:1 ratio for Panc08.13 cells. Gemcitabine and nab-paclitaxel were combined (nM:ng/mL) with a 1:1 ratio for HPAF-II cells and a 1:2 ratio for Panc08.13 cells. The drug treatment lasted for 4 days for HPAF-II, HPAF-II GATA6-knockout, Panc08.13, and Panc08.13 mCherry-expressing cells, but it lasted for 5 days for Panc08.13 GATA6-overexpressing cells to compensate for the reduced proliferation rate due to GATA6 overexpression. After drug treatment, 150 μL media was removed from each well, and 50 μL CellTiter-Glo (Promega, G7571) working solution was added to each well, followed by incubation and luminescence reading with a Tecan Infinite M200 plate reader according to the manufacturer’s protocol. IC_50_ values of each drug were calculated using GraphPad Prism 10. To examine the synergy between oxaliplatin and trametinib or MRTX1133 in HPAF-II and GATA6-knockout cells, drug combination studies were performed following the Chou-Talalay method (80) using the above-described cell viability assay and drug doses described in Supplemental Table 6.

### Bioinformatic analysis.

Bulk RNA-seq data of resected human pancreatic tumors or patient-derived PDAC xenografts were from the International Cancer Genome Consortium (ICGC) study (31), GSE252909 (32), and GSE215307 (33). The normalized gene expression data (cpm) were extracted from the original studies if provided. Otherwise, the sequencing reads were mapped to human genome (hg38) and mouse genome (mm39) using STAR aligner version 2.7.10b (81). Disambiguate algorithm (82) was used to assign reads to human or mouse genome, followed by gene expression quantification using featureCounts version 2.0.6 (83). In each cohort, genes without at least 1 CPM in 20% of samples were excluded from downstream analysis. *z* scores were calculated for each gene based on log_2_-transformed CPM values. The med_rank_siKRAS_ERKi_KRASi_UP (UP) and med_rank_siKRAS_ERKi_KRASi_DN (DN) gene signatures (29) were used to calculate the KRAS/ERK activity score. The KRAS/ERK activity score = the average of *z* scores of genes in the UP gene signature − the average of *z* scores of genes in the DN gene signature.

scRNA-seq data of resected human pancreatic tumors and control pancreases were from CRA001160 in the Genome Sequence Archive (34). Normal ductal cells and malignant cells were extracted from the scRNA-seq dataset. The KRAS/ERK activity scores were calculated for each cell using the AddModuleScore function in Seurat (84) using the above-mentioned gene signatures. The FetchData function in Seurat was used to extract the *GATA6* expression levels.

### Statistics.

Statistical analysis was performed using GraphPad Prism 10 or as indicated. A *P* value of less than 0.05 was considered significant. Statistical tests are described in figure legends where applicable.

### Study approval.

The SingHealth IACUC approved all animal studies.

### Data availability.

The raw Cut&Run-seq data have been deposited in the National Center for Biotechnology Information’s Gene Expression Omnibus database (GSE307506). The public transcriptomic datasets used in this study were from GSE58055 (27), GSE201412 (28), GSE248058 (30), the ICGC study (31), GSE252909 (32), GSE215307 (33), and CRA001160 (34). The genome-wide CRISPR screen results are shown in Supplemental Table 1. Values underlying graphed data in main and supplemental figures are shown in the Supporting Data Values file. Uncropped blot images are provided as a supplemental file.

## Author contributions

ZZ and DMV designed the study. ZZ and PJL performed the biochemical assays. ZZ, XC, RS, JC, and YW performed the bioinformatics analyses. JAK, CAS, and CJD contributed the ERK-regulated proteomics dataset. ZZ and DMV wrote the manuscript, which was reviewed, revised, and approved by all of the authors.

## Funding support

This work is the result of NIH funding, in whole or in part, and is subject to the NIH Public Access Policy. Through acceptance of this federal funding, the NIH has been given a right to make the work publicly available in PubMed Central.

- National Research Foundation Singapore.
- Ministry of Health’s National Medical Research Council under Singapore Translational Research (STaR) award MOH-000155 (to DMV).
- National Cancer Institute grants R01CA42978, P50CA196510, P50CA257911, U01CA199235, P01CA203657, and R35CA232113 (to CJD).
- Pancreatic Cancer Action Network/American Association for Cancer Research (22-WG-DERB) (to CJD).
- Department of Defense (W81XWH2110692) (to CJD).

## Supplementary Material

Supplemental data

Unedited blot and gel images

Supplemental table 1

Supplemental tables 2-6

Supporting data values

## Version 1 12/02/2025

In-Press Preview

## Version 2 02/02/2026

Electronic publication
