# Supplementary material for: Oncogenic KRAS/ERK/JUNB signaling suppresses differentiation regulator GATA6 in pancreatic cancer
Source: J Clin Invest. 2025 Dec 2;136(3):e191370. doi: 10.1172/JCI191370 (PMC12867130; doi:10.1172/JCI191370)
Supplement: Supplemental data [file jci-136-191370-s209.pdf]

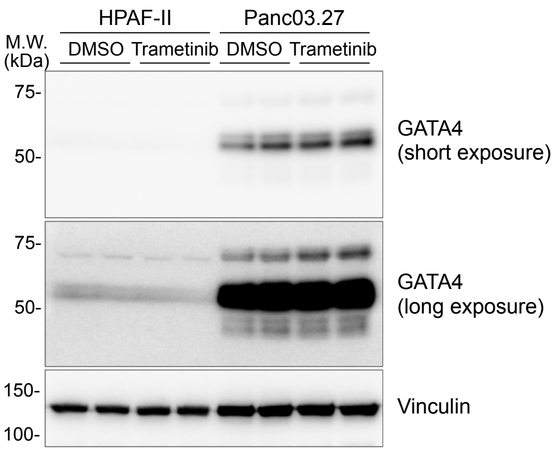

Supplementary Figure S1. MEK inhibition did not significantly increase GATA4 protein abundance in HPAF-II and Panc03.27 cells. HPAF-II and Panc03.27 cells were treated with 100 nM trametinib or DMSO control for 24 h, followed by Western blot analysis. Vinculin was used as loading control. Each lane represents a biological replicate. n = 2 biological replicates/condition.

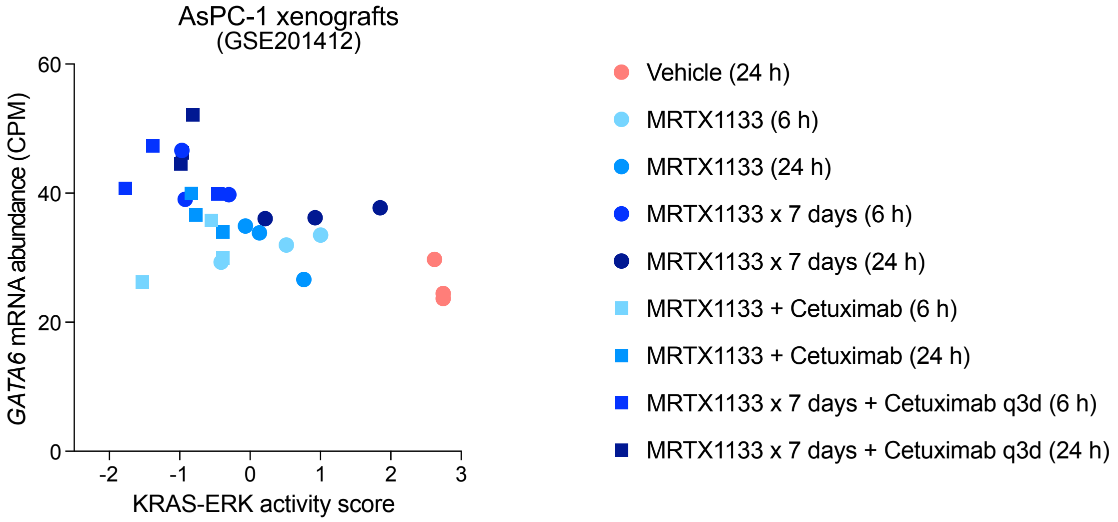

Supplementary Figure S2. KRAS inhibition up-regulated *GATA6* mRNA abundance in AsPC-1 xenografts.
Analysis of data from GSE201412[28]. Mice bearing AsPC-1 xenografts were treated with vehicle or the KRAS^{G12D} inhibitor MRTX1133 alone or in combination with cetuximab (monoclonal antibody targeting EGFR). Tumors were harvested at the indicated time points.

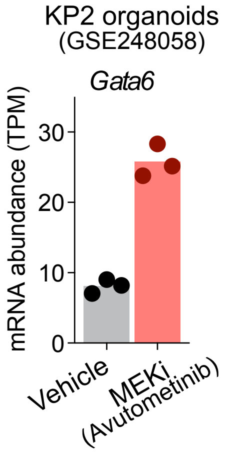

Supplementary Figure S3. MEK inhibition up-regulated *Gata6* mRNA abundance in KP2 mouse pancreatic cancer organoids. Analysis of data from GSE248058[30]. KP2 mouse pancreatic cancer organoids (p48-Cre; LSL-*Kras*^{G12D}; *Trp53*$^{flox/flox}$) were treated with vehicle or MEK inhibitor avutometinib (100 nM) for 24 h.

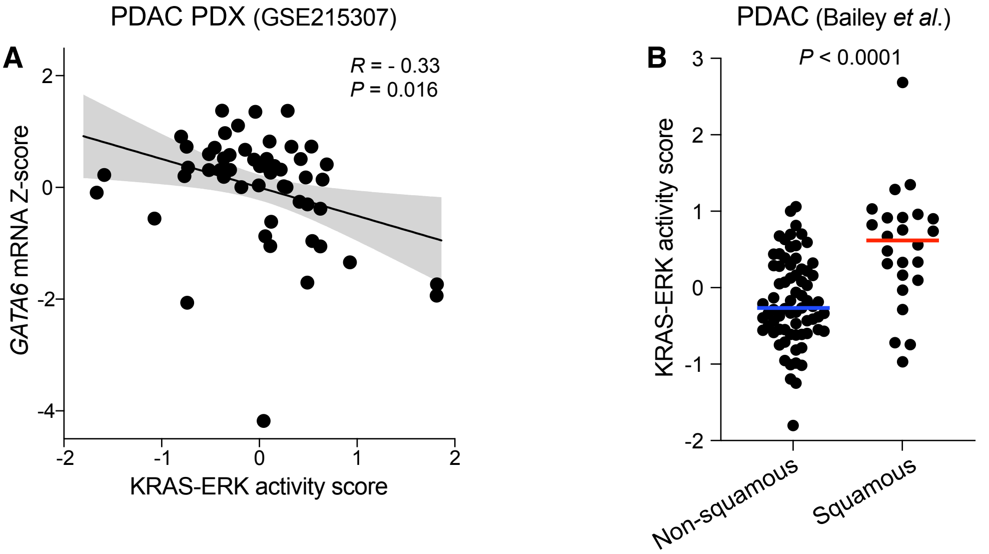

Supplementary Figure S4. *GATA6* expression negatively correlated with KRAS/ERK activity in pancreatic tumors.

(**A**) *GATA6* mRNA abundance negatively correlated with KRAS/ERK activity in a cohort of pancreatic cancer patient-derived tumor xenografts (PDAC PDX). Analysis of RNA-seq data from GSE215307[33]. Each dot represents an individual PDX. Only human transcripts from the PDX (hence originating from the malignant cells) were analyzed. The *R* and *P* values of simple linear regression are shown.

(**B**) Pancreatic tumors of the squamous subtype showed higher KRAS/ERK activity compared to non-squamous pancreatic tumors. Analysis of data from the Bailey cohort[31]. Each dot represents a human primary pancreatic tumor. The *P* value of two-tailed, unpaired *t* test is shown.

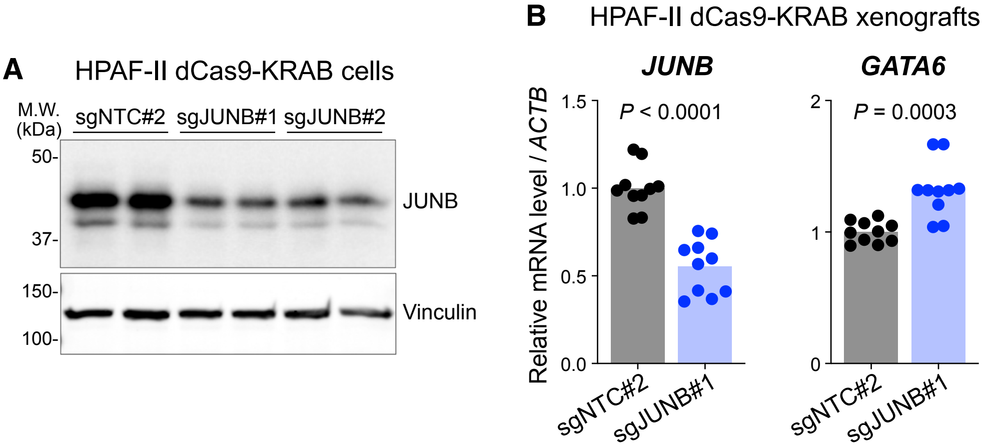

Supplementary Figure S5. Knockdown of *JUNB* increased *GATA6* transcript abundance in vivo.

(**A**) Knockdown of *JUNB* by CRISPRi decreased JUNB protein abundance. HPAF-II cells stably expressing dCas9-KRAB fusion protein were transduced with a lentivirus expressing a doxycycline inducible non-targeting control sgRNA (sgNTC#2) or a sgRNA targeting the *JUNB* promoter (sgJUNB#1 or #2). Cells were treated with 1 μg/ml doxycycline for six days and then analyzed by Western blot. *n* = 2 biological replicates per condition.

(**B**) Knockdown of *JUNB* increased *GATA6* transcript abundance in HPAF-II xenografts. HPAF-II cells from (**A**), above, were implanted subcutaneously in NSG mice. After tumor establishment, mice were fed ad libitum with standard diet containing 600 mg/kg doxycycline for nine days. Tumors were harvested and analyzed by RT-qPCR. *n* = 5 tumors per group, each analyzed with technical replicates. The *P* values of two-tailed, unpaired *t* test are shown.

CPTAC pancreatic cancer cohort

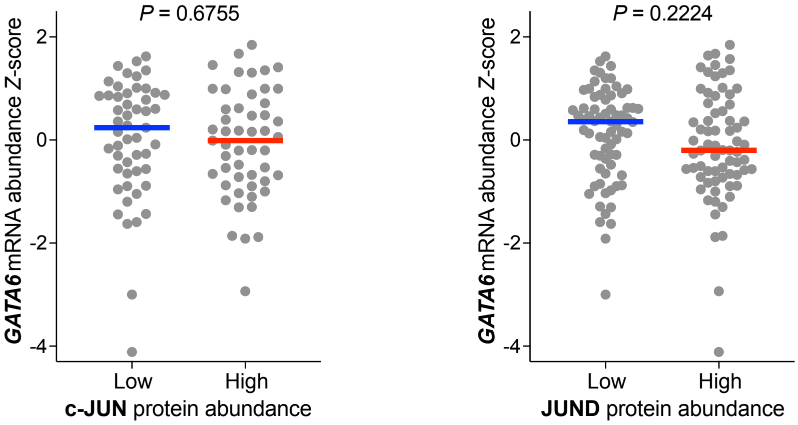

Supplementary Figure S6. Protein abundance of c-JUN or JUND did not correlate to *GATA6* mRNA abundance.

Protein abundance of c-JUN and JUND and mRNA abundance of *GATA6* in human pancreatic tumors in the CPTAC cohort[39] were obtained from the cBioPortal website[84]. Each dot represents an individual tumor and mean values of each group are shown. *n* = 51/group for c-JUN and 70/group for JUND. *P* values of two-tailed, unpaired *t* test are shown.

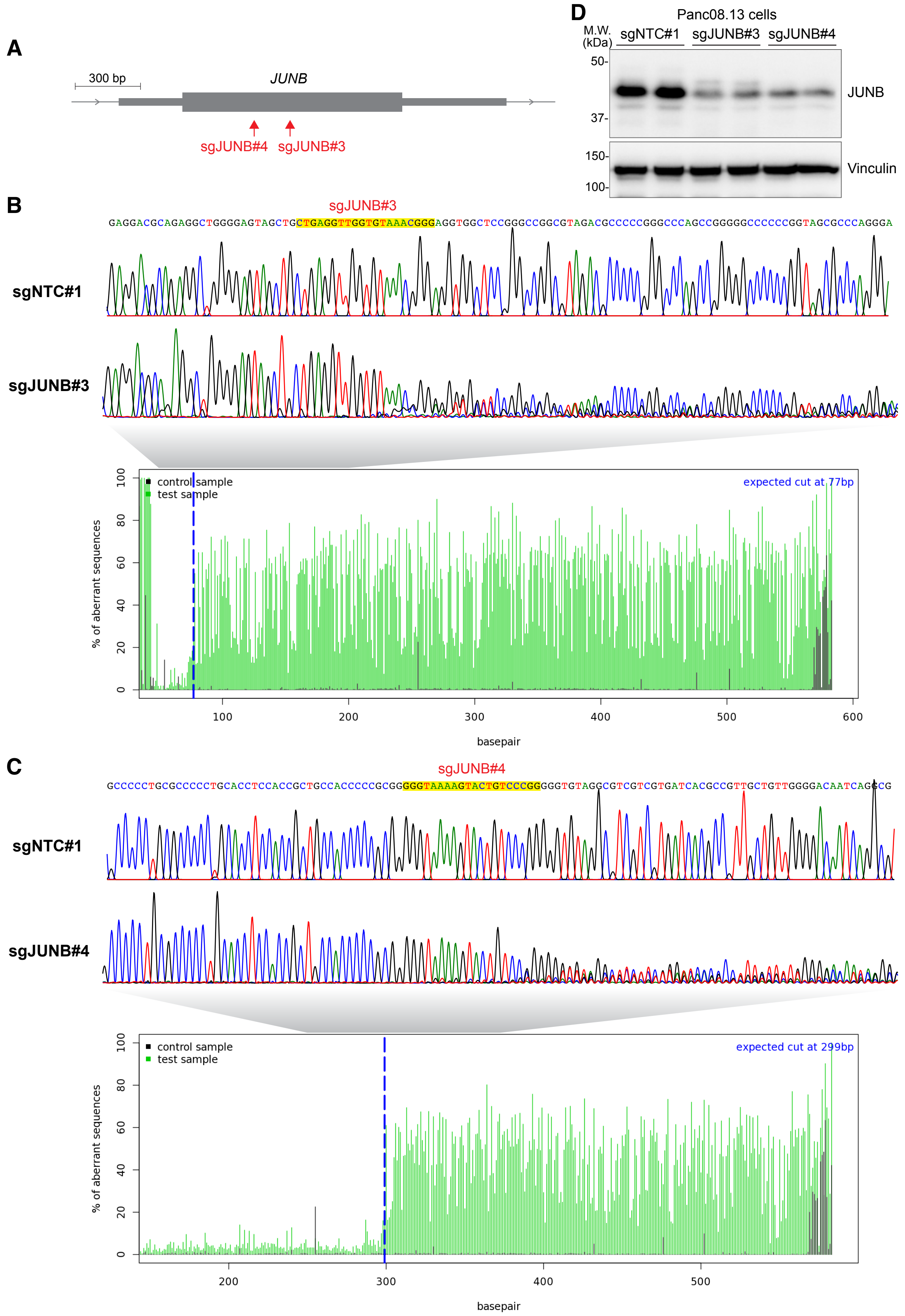

Supplementary Figure S7. CRISPR-mediated *JUNB* knockout in Panc08.13 cells.
(**A**) Two individual sgRNAs (sgJUNB#3 and #4) were used to knockout *JUNB*. The genome positions targeted are indicated with arrows.
(**B** and **C**) sgJUNB#3 (**B**) and sgJUNB#4 (**C**) led to a considerable number of indels at the expected cutting sites on the genome. Genomic DNA was extracted from Panc08.13 cells transduced with lentivirus packaging a non-targeting control sgRNA (sgNTC#1), sgJUNB#3, or sgJUNB#4, followed by PCR amplification with primers flanking the expected cutting sites and Sanger sequencing. In each panel, the reference DNA sequence is shown on the top with the sgRNA sequence highlighted in yellow. Sanger sequencing traces are shown in the middle and their comparison via TIDE[68] is shown at the bottom.
(**D**) The *JUNB* knockout cell pools were analyzed by Western blotting. n = 2 biological replicates per condition.

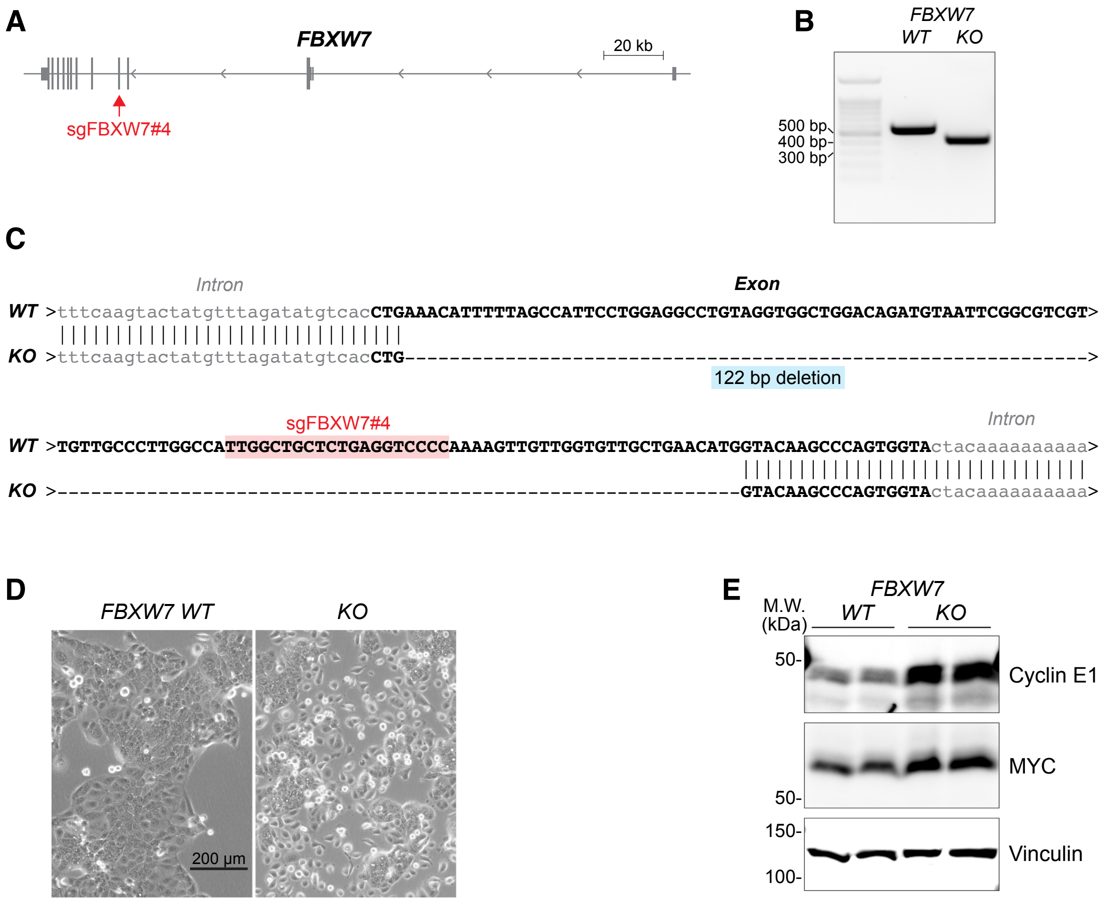

Supplementary Figure S8. Characterization of a *FBXW7* homozygous knockout clone of HPAF-II cells.

(**A**) sgFBXW7#4 was used to knockout *FBXW7* in HPAF-II cells. The exon targeted by sgFBXW7#4 is indicated with the arrow.

(**B** and **C**) Establishment of a *FBXW7* homozygous knockout (KO) clone with a 122 bp deletion. The *FBXW7* wildtype (WT) HPAF-II cells were transduced with a non-targeting control sgRNA. Genomic DNA was extracted, followed by PCR amplification with primers flanking the expected cutting sites. Agarose gel electrophoresis results (**B**) and Sanger sequencing traces (**C**) for the PCR amplicons are shown.

(**D** and **E**) *FBXW7* knockout changed HPAF-II cell morphology (**D**) and increased protein abundance of known FBXW7 substrates Cyclin E and MYC (**E**).

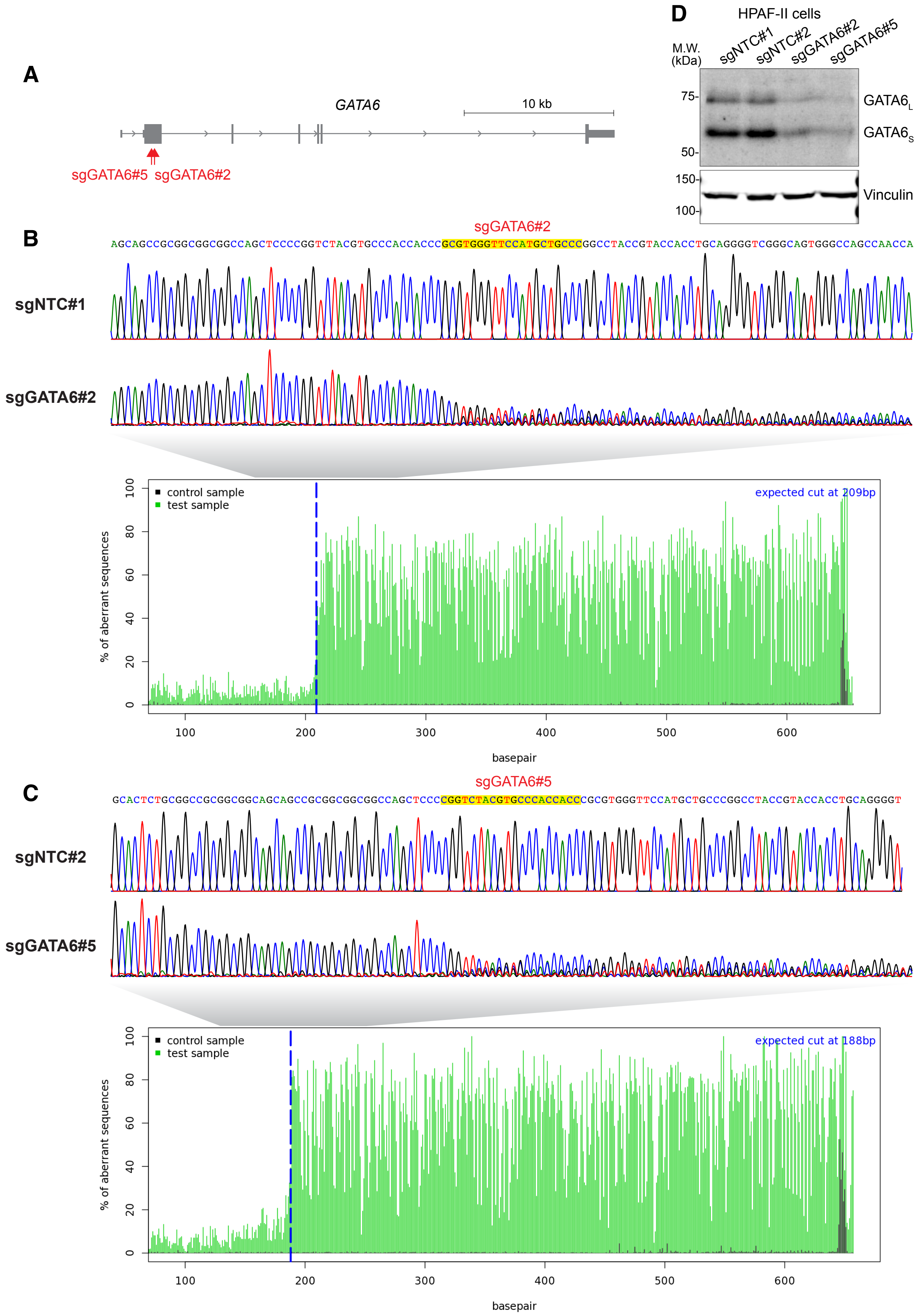

Supplementary Figure S9. CRISPR-mediated *GATA6* knockout in HPAF-II cells.
(**A**) Two individual sgRNAs (sgGATA6#2 and #5) were used to knockout *GATA6*. The exon targeted is indicated with arrows.
(**B** and **C**) sgGATA6#2 (**B**) and sgGATA6#5 (**C**) led to a considerable number of indels at the expected cutting sites on the genome. Genomic DNA was extracted from HPAF-II cells transduced with lentivirus packaging a non-targeting control sgRNA (sgNTC#1 or #2), sgGATA6#2, or sgGATA6#5, followed by PCR amplification with primers flanking the expected cutting sites and Sanger sequencing. In each panel, the reference DNA sequence is shown on the top with the sgRNA sequence highlighted in yellow. Sanger sequencing traces are shown in the middle and their comparison via TIDE[68] is shown at the bottom.
(**D**) The *GATA6* knockout cell pools were analyzed by Western blotting.

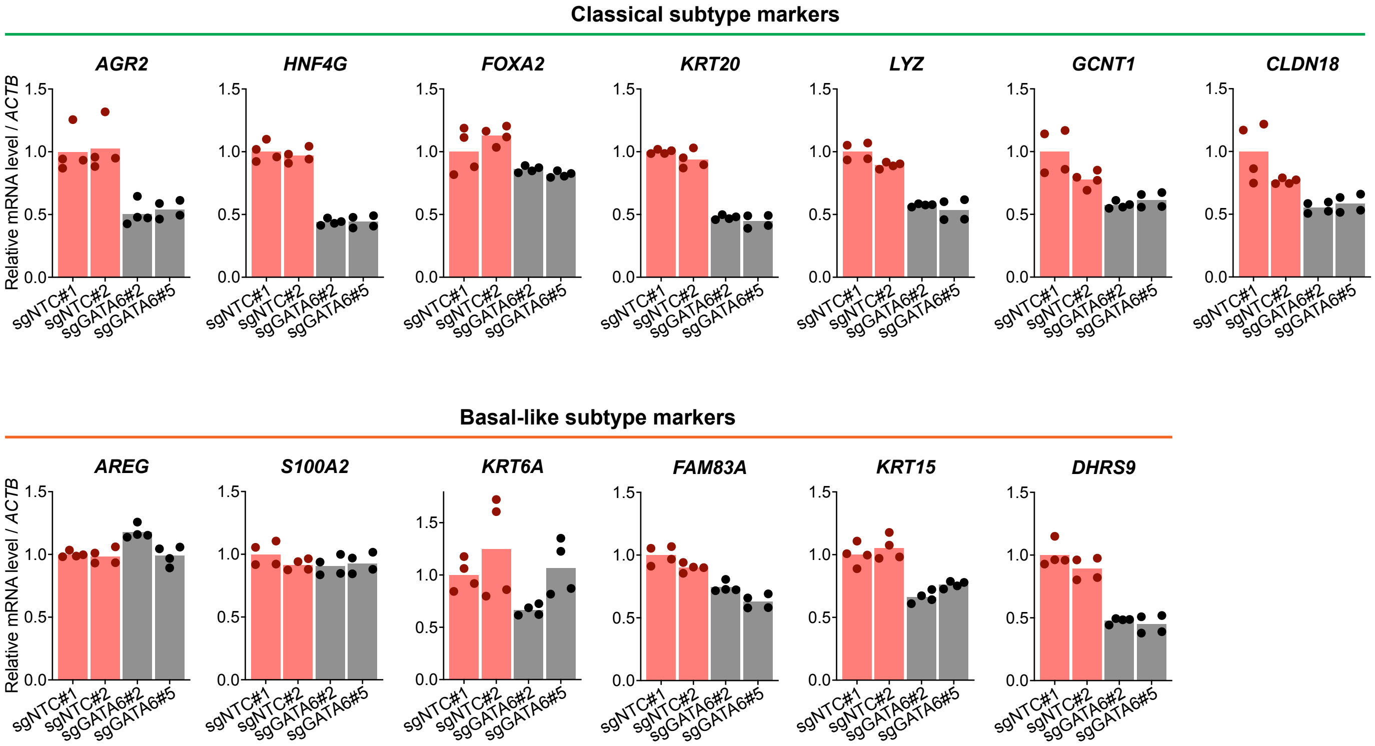

Supplementary Figure S10. ***GATA6*** **knockout down-regulated classical subtype genes in HPAF-II cells.**
The two HPAF-II cell pools shown in **Supplementary Figure S9** were analyzed by RT-qPCR for classical or basal-like subtype markers. *n* = 2 biological replicates × 2 technical replicates per condition.

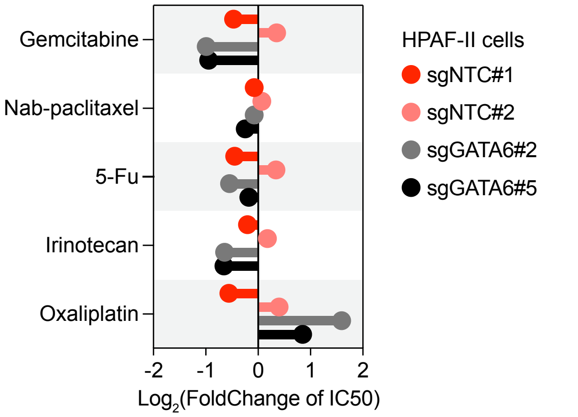

Supplementary Figure S11. ***GATA6*** **knockout conferred resistance to oxaliplatin in HPAF-II cells.** Responses to individual chemotherapy agents were examined for the HPAF-II cell pools shown in **Supplementary Figure S9**. Changes in IC50 values relative to the average of sgNTC#1 and sgNTC#2 are shown.

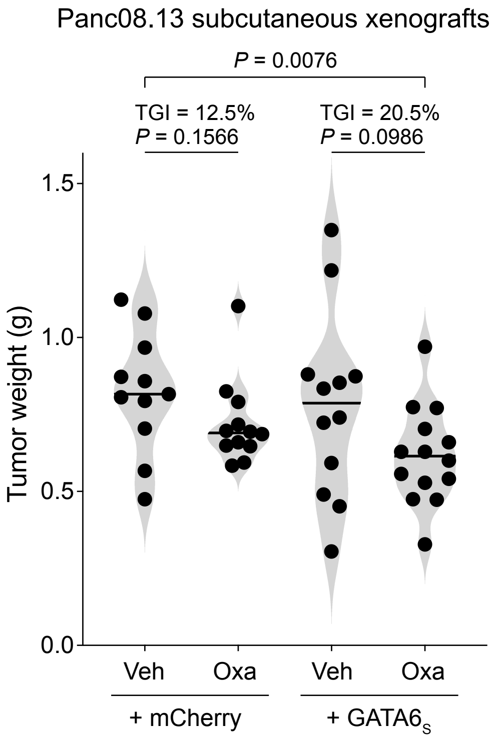

Supplementary Figure S12. *GATA6* overexpression sensitized Panc08.13 tumors to oxaliplatin.
NSG mice bearing Panc08.13 xenografts that overexpressed $GATA6_S$ or mCherry control were treated with vehicle (Veh) or oxaliplatin (Oxa, 5 mg/kg per intraperitoneal injection, twice weekly) for two weeks. *n* = 11-14 tumors per condition. *P* values of two-tailed, unpaired *t* test are shown. TGI, tumor growth inhibition ratio.

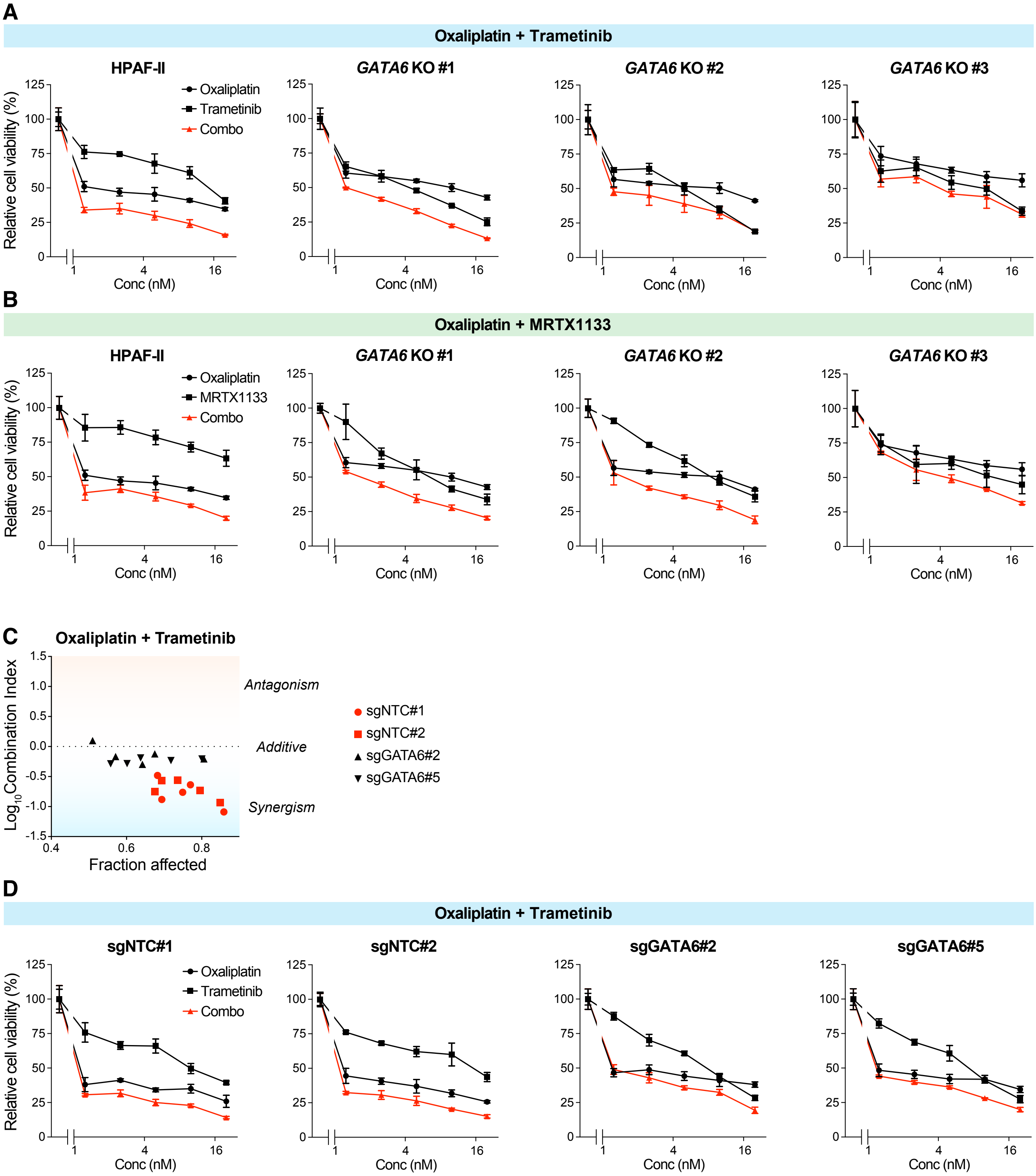

Supplementary Figure S13. Combination of oxaliplatin and KRAS/ERK inhibitors in HPAF-II cells and *GATA6* knockout cells.

(**A** and **B**) Dose response curves of HPAF-II cells and *GATA6* knockout clonal lines to oxaliplatin, trametinib, MRTX1133, and drug combinations. Cells were treated as described in Methods. The concentration (Conc) shown in x-axes of these plots refers to the concentration of trametinib or MRTX1133 in each condition (the concentration of oxaliplatin was linked to trametinib/MRTX1133 as shown in **Supplementary Table S6**). *n* = 2-3 biological replicates/condition. The drug combination indices calculated with the Chou-Talalay method[80] are shown in **Figure 9A**.

(**C** and **D**) Drug combination studies in HPAF-II cell pools transduced with sgNTC#1/2 and sgGATA6#2/5 (**Supplementary Figure S9**) showed similar results as in the HPAF-II parental cells and *GATA6* knockout clonal lines (**Figure 9A** and **Supplementary Figure S13A** and **B**).

sgGATA6#KI | Destroy sgGATA6#KI site in repair template | 150 bp homology arm | T2A

```
GCACCAGGAATCCGGTCGCACGGAGGGTGTCCCCCTCTTCTGCCAGGCGGGTGCCCCGGTGATGACTGGTGCGGGAGAGA
GCACCAATCCCGAGAACAGCGAGCTCAAGTATTCGGGTCAAGATGGGCTCTACATAGGCGTCAGTCTCGCCTCGCCGGCC
GAAGTCACGTCCTCtGTGCGcCCGGAcTCCTGGTGCGCCCTGGCCCTGGCCgggtccggtgagggcagaggaagtctgct
aacatgcggtgacgtcgaggagaatcctggcccaatggtgagcaagggcgaggagctgttcaccggggtggtgcccatcc
tggtcgagctggacggcgacgtaaacggccacaagttcagcgtgtccggcgagggcgagggcgatgccacctacggcaag
ctgaccctgaagttcatctgcaccaccggcaagctgcccgtgccctggcccaccctcgtgaccaccctgacctacggcgt
gcagtgcttcagccgctaccccgaccacatgaagcagcacgacttcttcaagtccgccatgcccgaaggctacgtccagg
agcgcaccatcttcttcaaggacgacggcaactacaagacccgcgccgaggtgaagttcgagggcgacaccctggtgaac
cgcatcgagctgaagggcatcgacttcaaggaggacggcaacatcctggggcacaagctggagtacaactacaacagcca
caacgtctatatcatggccgacaagcagaagaacggcatcaaggtgaacttcaagatccgccacaacatcgaggacggca
gcgtgcagctcgccgaccactaccagcagaacacccccatcggcgacggccccgtgctgctgcccgacaaccactacctg
agcacccagtccgccctgagcaaagaccccaacgagaagcgcgatcacatggtcctgctggagttcgtgaccgccgccgg
gatcactctcggcatggacgagctgtacaagtaaTGCGACCGGATTCCTGGTGCGCCCTGGCCCTGGCCTGAGCCCACGC
CGCCAGGAGGCAGGGAGGGCTCCGCCGCGGGCCTCACTCCACTCGTGTCTGCTTTTGTGCAGCGGTCCAGACAGTGGCGA
CTGCGCTGACAGAACGTGATTCTCCCTCCGTGCGACCGGATTCCTGGTGC
```

Stop codon | sgGATA6#KI | EGFP | 150 bp homology arm

sgJUNB#KI | linker | 150 bp homology arm | EGFP | IRES

```
AGGGGCAGGGGACGTTCAGAAGGGACAAGGTGAAGACGCTCAAGGCCGAGAACGCGGGGCTGTCGAGTACCGCCGGCCTC
CTCCGGGAGCAGGTGGCCCAGCTCAAACAGAAGGTCATGACCCACGTCAGCAACGGCTGTCAGCTGCTGCTTGGGGTCAA
GGGACACGCCTTCGGATCTGCCGGGTCTGCTGCTGGTAGCGGTGAGTTCgtgagcaagggcgaggagctgttcaccgggg
tggtgcccatcctggtcgagctggacggcgacgtaaacggccacaagttcagcgtgtccggcgagggcgagggcgatgcc
acctacggcaagctgaccctgaagttcatctgcaccaccggcaagctgcccgtgccctggcccaccctcgtgaccaccct
gacctacggcgtgcagtgcttcagccgctaccccgaccacatgaagcagcacgacttcttcaagtccgccatgcccgaag
gctacgtccaggagcgcaccatcttcttcaaggacgacggcaactacaagacccgcgccgaggtgaagttcgagggcgac
accctggtgaaccgcatcgagctgaagggcatcgacttcaaggaggacggcaacatcctggggcacaagctggagtacaa
ctacaacagccacaacgtctatatcatggccgacaagcagaagaacggcatcaaggtgaacttcaagatccgccacaaca
tcgaggacggcagcgtgcagctcgccgaccactaccagcagaacacccccatcggcgacggccccgtgctgctgcccgac
aaccactacctgagcacccagtccgccctgagcaaagaccccaacgagaagcgcgatcacatggtcctgctggagttcgt
gaccgccgccgggatcactctcggcatggacgagctgtacaagtaaatgcatgagtaactgaggatccgcccctctccct
ccccccccctaacgttactggccgaagccgcttggaataaggccggtgtgcgtttgtctatatgttattttccaccata
ttgccgtcttttggcaatgtgagggcccggaaacctggccctgtcttcttgacgagcattcctaggggtctttcccctct
cgccaaaggaatgcaaggtctgttgaatgtcgtgaaggaagcagttcctctggaagcttcttgaagacaaacaacgtctg
tagcgaccctttgcaggcagcggaaccccccacctggcgacaggtgcctctgcggccaaaagccacgtgtataagataca
cctgcaaaggcggcacaaccccagtgccacgttgtgagttggatagttgtggaaagagtcaaatggctctcctcaagcgt
attcaacaaggggctgaaggatgcccagaaggtaccccattgtatgggatctgatctggggcctcggtacacatgcttta
catgtgtttagtcgaggttaaaaaaacgtctaggccccccgaaccacggggacgtggttttcctttgaaaaacacgatga
taatatggccacaaccctggaattcgccaccatggtgagcaagggcgaggaggataacatggccatcatcaaggagttca
tgcgcttcaaggtgcacatggagggctccgtgaacggccacgagttcgagatcgagggcgagggcgagggccgcccctac
gagggcacccagaccgccaagctgaaggtgaccaagggtggccccctgcccttcgcctgggacatcctgtcccctcagtt
catgtacggctccaaggcctacgtgaagcaccccgccgacatccccgactacttgaagctgtccttccccgagggcttca
agtgggagcgcgtgatgaacttcgaggacggcggcgtggtgaccgtgacccaggactcctccctgcaggacggcgagttc
atctacaaggtgaagctgcgcggcaccaacttcccctccgacggccccgtaatgcagaagaagaccatgggctgggaggc
ctcctccgagcggatgtaccccgaggacggcgccctgaagggcgagatcaagcagaggctgaagctgaaggacggcggcc
actacgacgctgaggtcaagaccacctacaaggccaagaagcccgtgcagctgcccggcgcctacaacgtcaacatcaag
ttggacatcacctcccacaacgaggactacaccatcgtggaacagtacgaacgcgccgagggccgccactccaccggcgg
catggacgagctgtacaagtaaACGTCCCCTGCCCCTTTACGGACACCCCCTCGCTTGGACGGCTGGGCACACGCCTCCC
ACTGGGGTCCAGGGAGCAGGCGGTGGGCACCCACCCTGGGACCTAGGGGCGCCGCAAACCACACTGGACTCCGGCCCTCC
TACCCTGCGCCCTCTGAACGTCCCCTGCCCCT
```

sgJUNB#KI | mCherry | 150 bp homology arm

Supplementary Figure S14. Repair templates for CRISPR-mediated gene knockin. Synthesized DNA sequences used for knocking fluorescence reporter cassettes into *GATA6* or *JUNB* loci are shown and annotated.
