# Supplementary material for: Oncogenic KRAS/ERK/JUNB signaling suppresses differentiation regulator GATA6 in pancreatic cancer
Source: J Clin Invest. 2025 Dec 2;136(3):e191370. doi: 10.1172/JCI191370 (PMC12867130; doi:10.1172/JCI191370)

Figure 1A

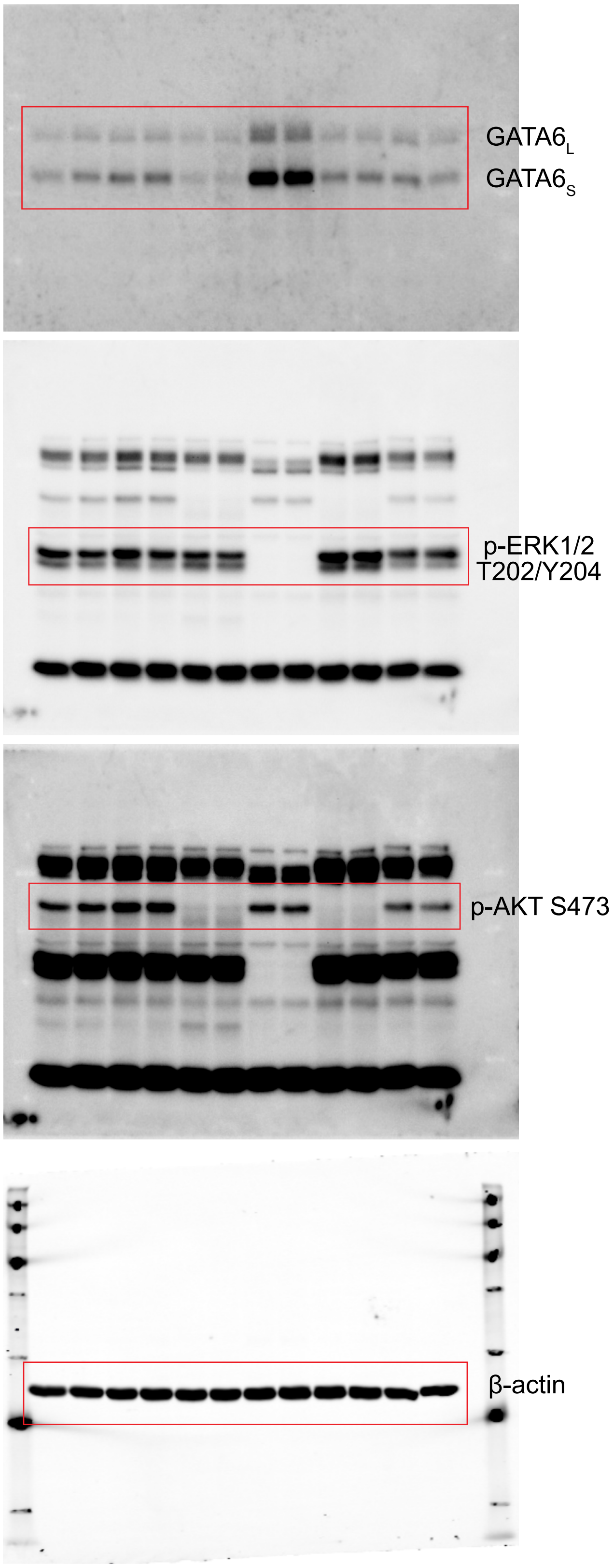

Figure 1B

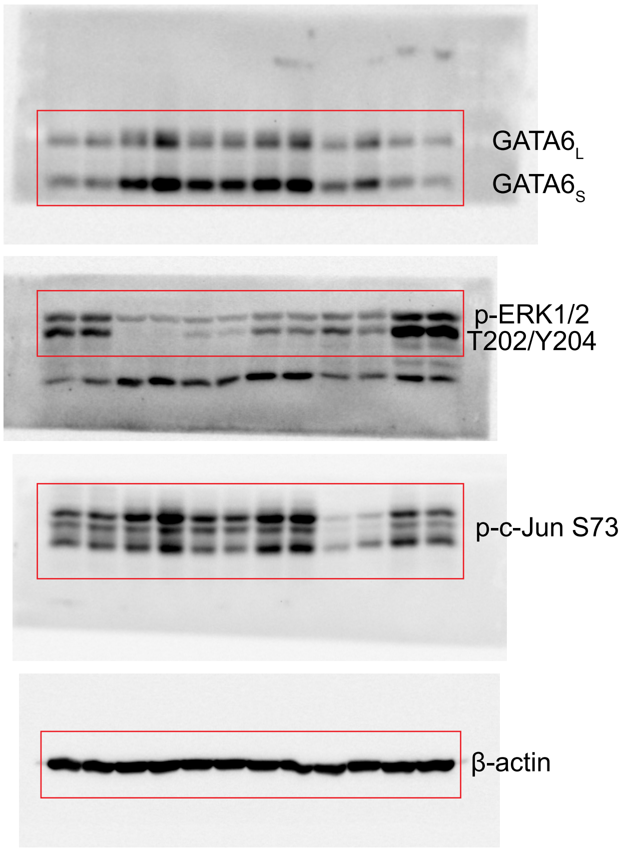

Figure 1C

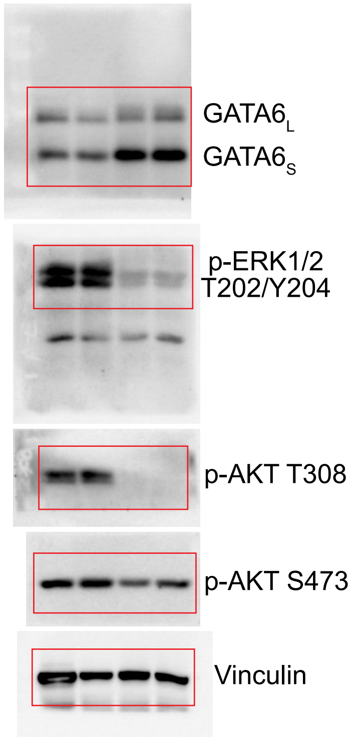

Figure 1D

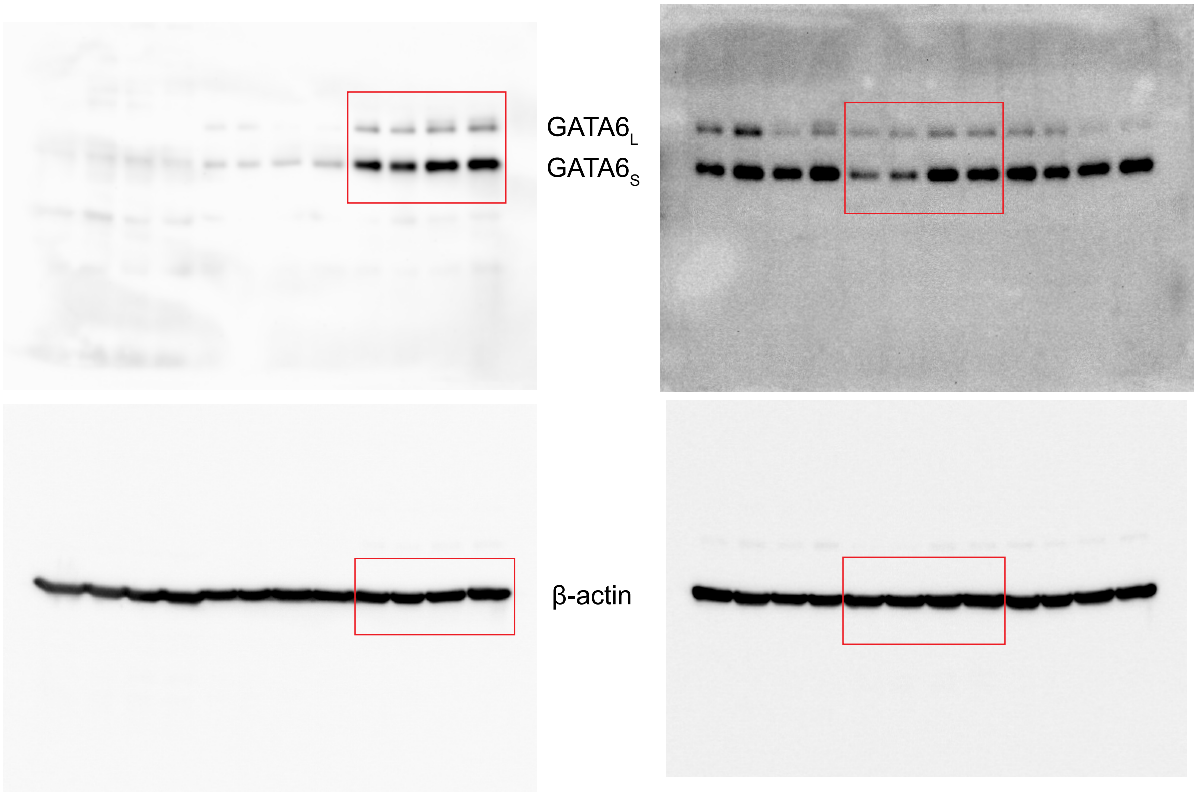

Figure 2A

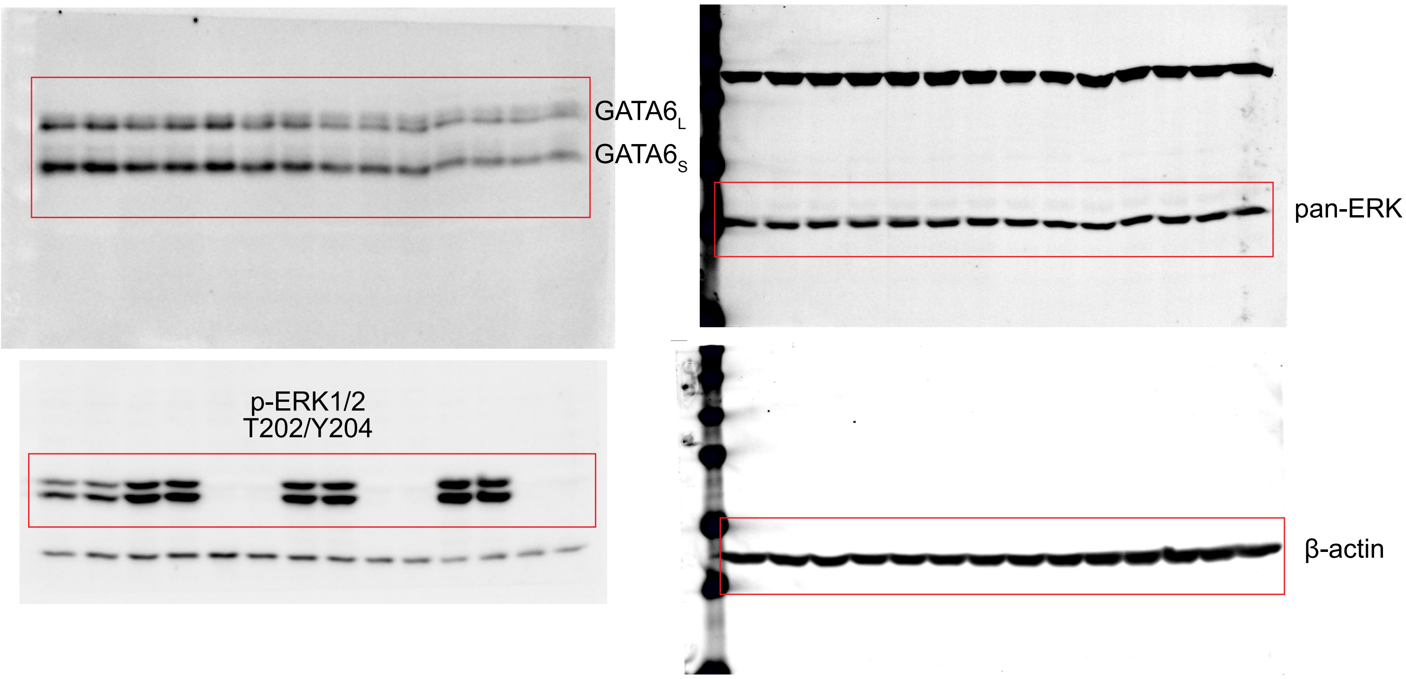

Figure 3A

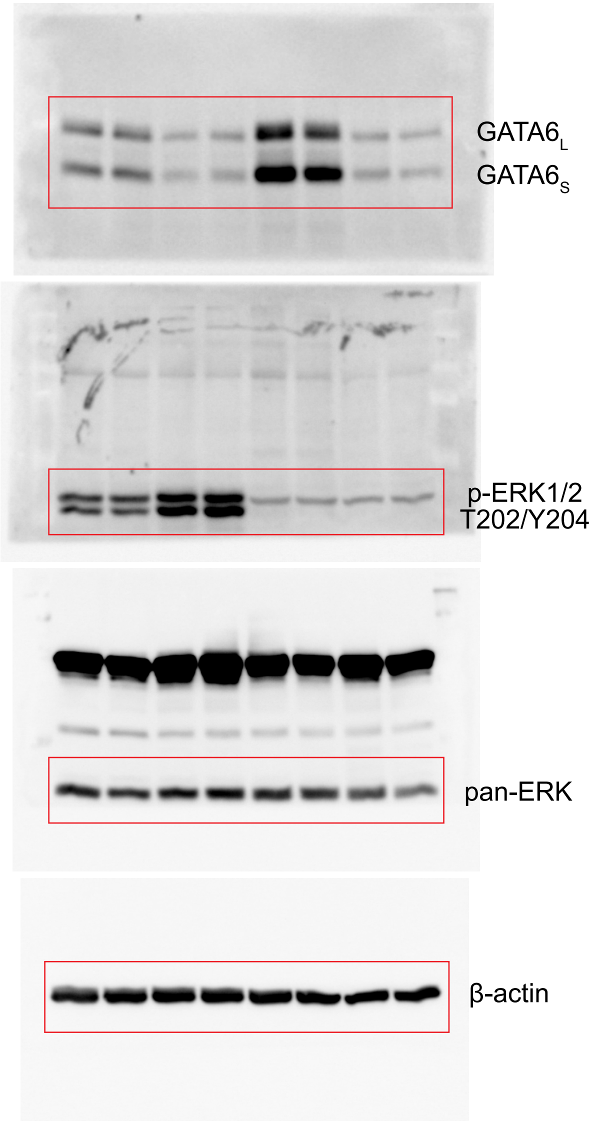

Figure 5D

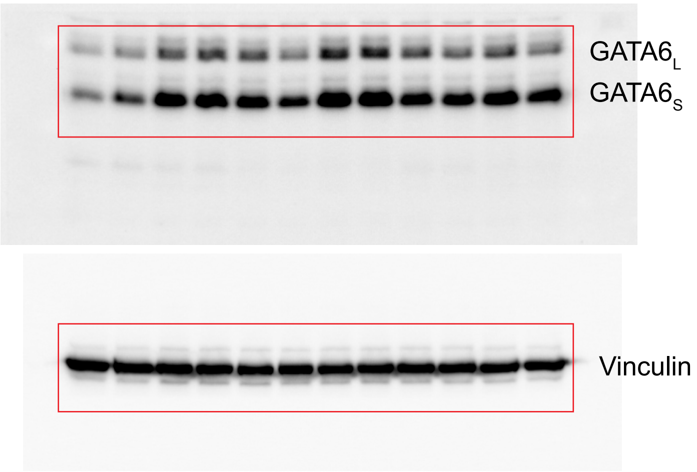

Figure 5E

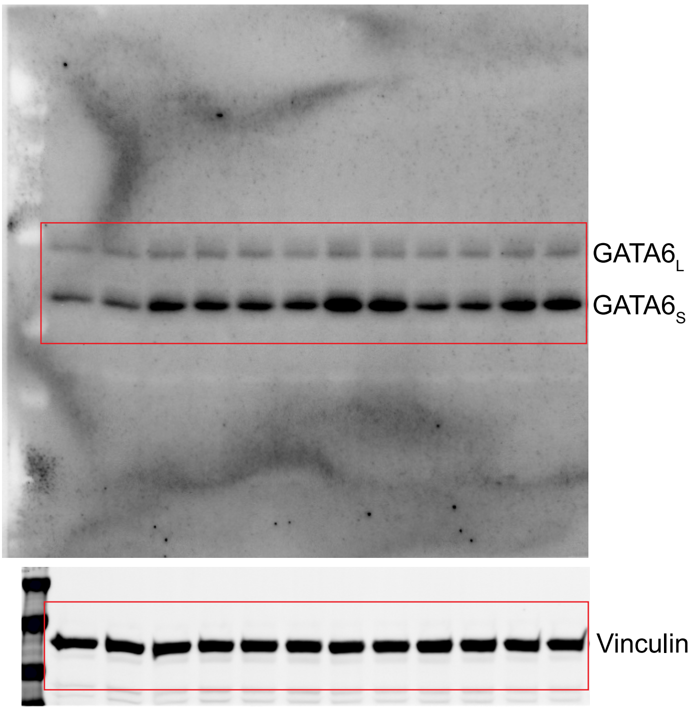

Figure 7C

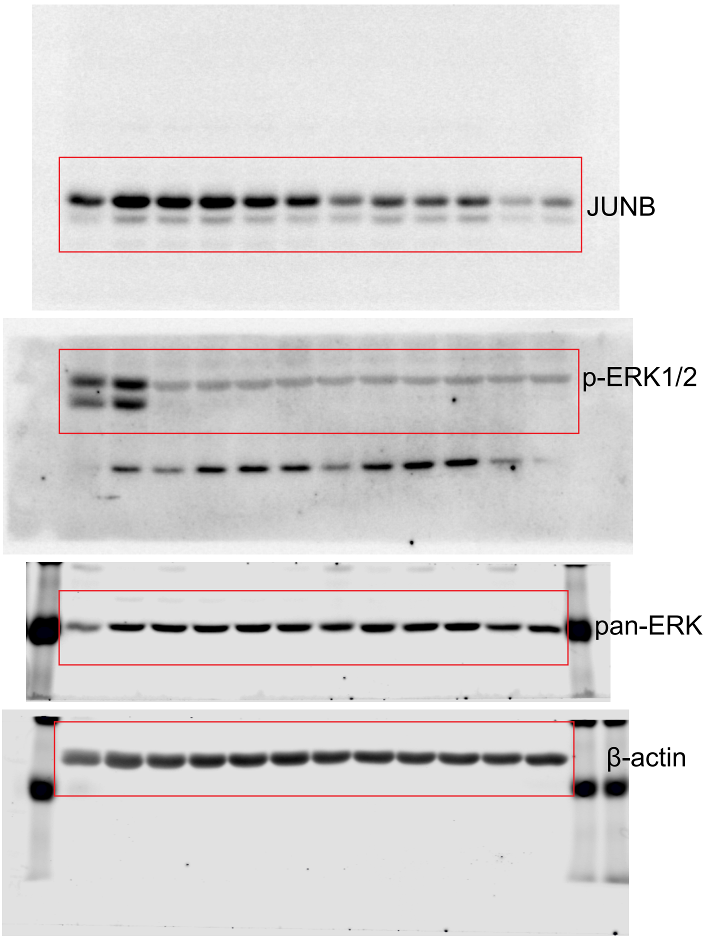

Figure 7E

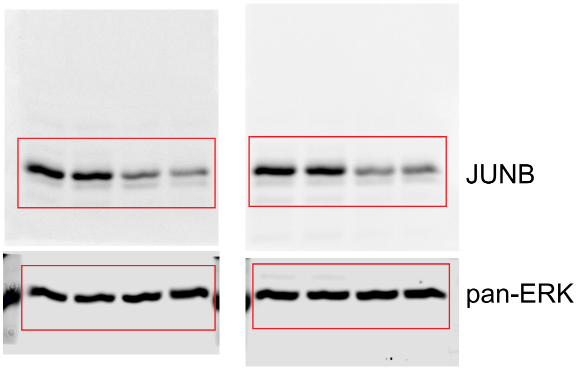

Figure 7F

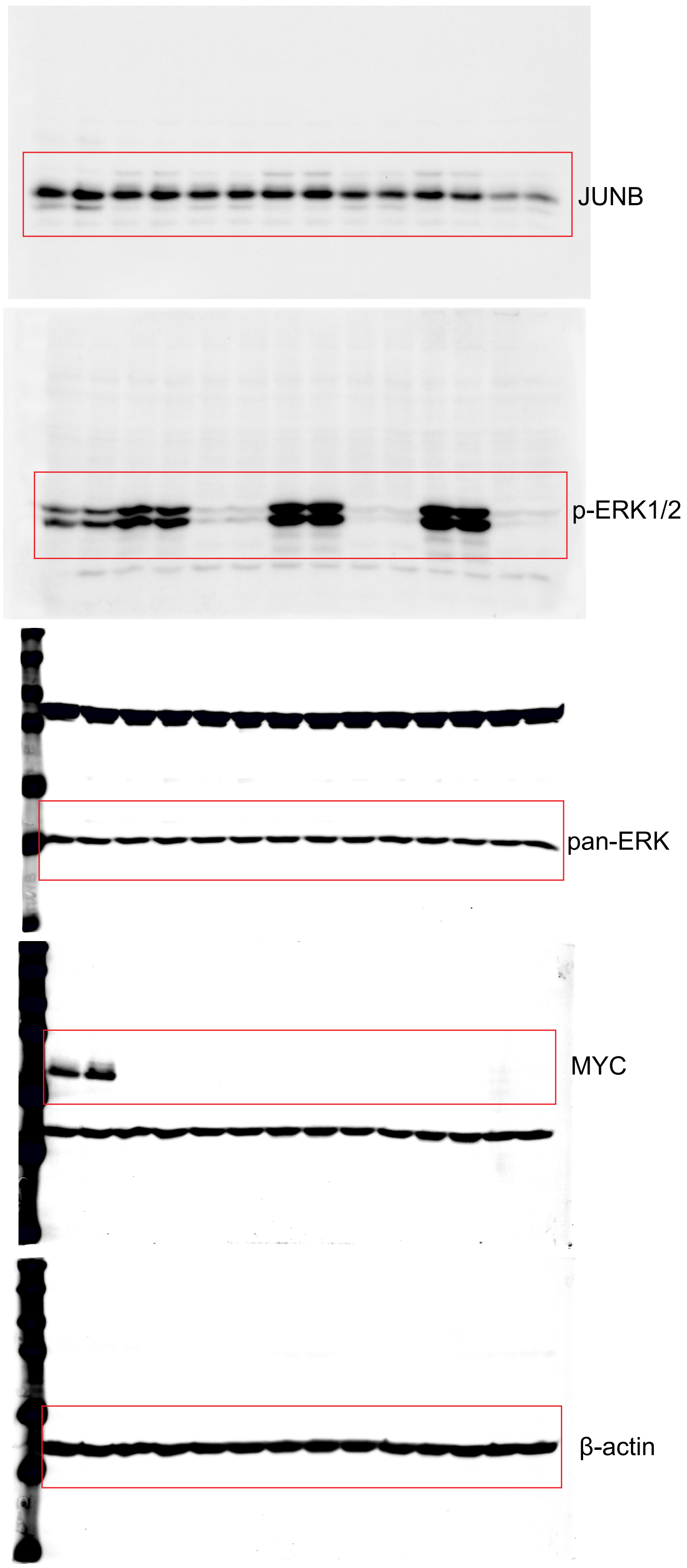

Figure 7I

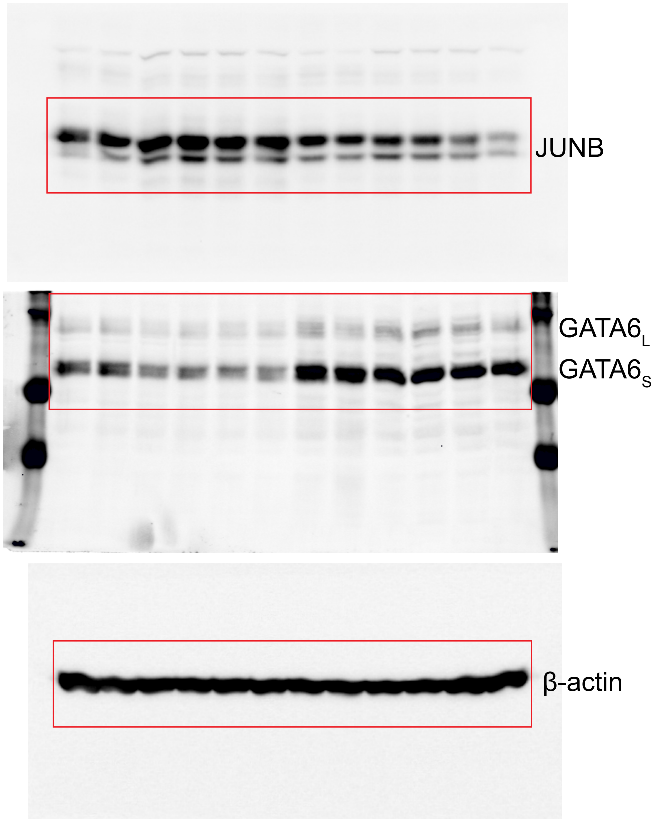

Figure 7J

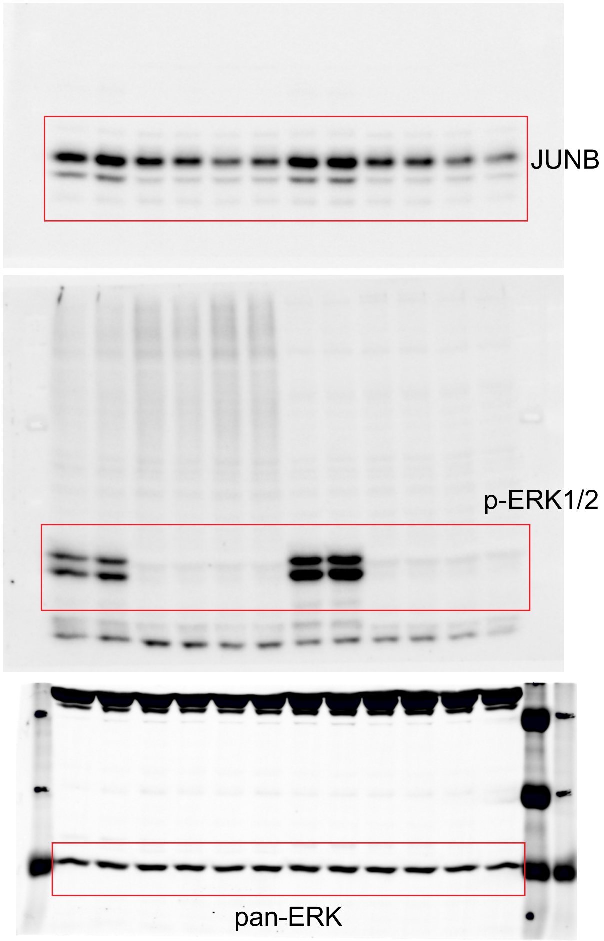

Figure 8A

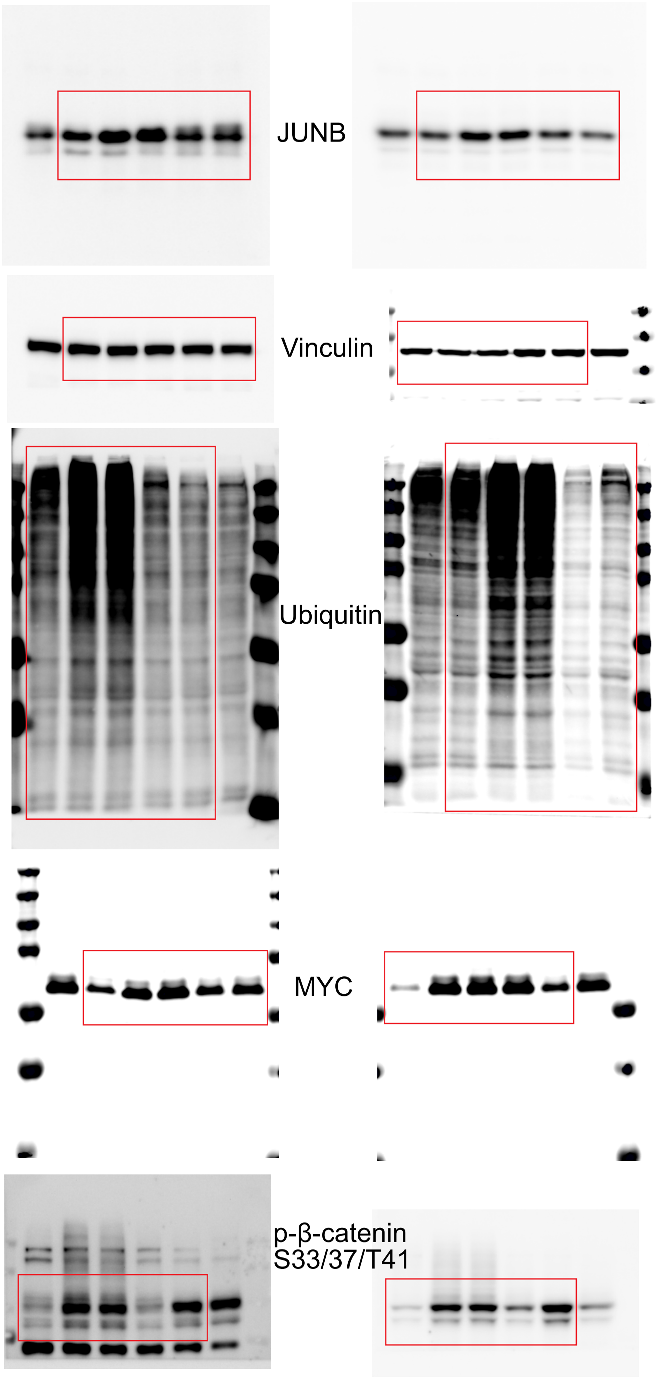

Figure 8B

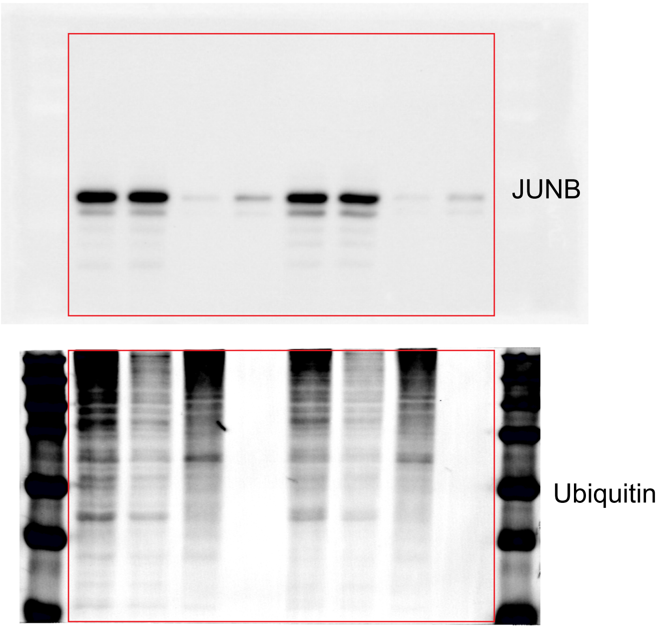

Figure 8C

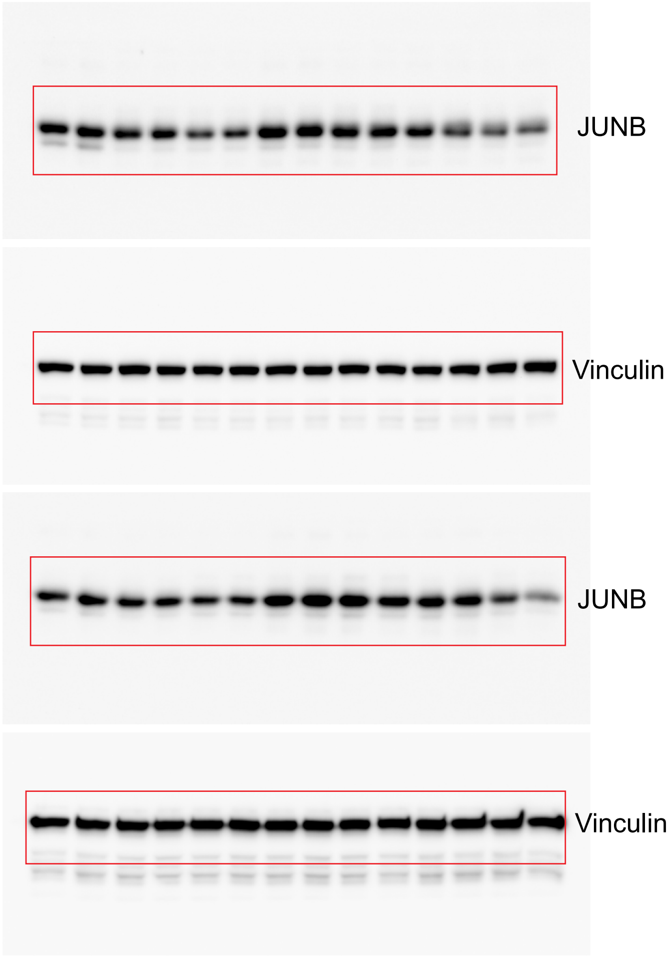

Figure 9A

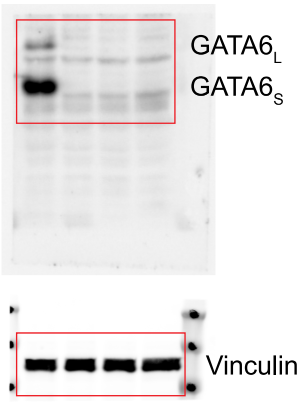

Supplementary Figure S1

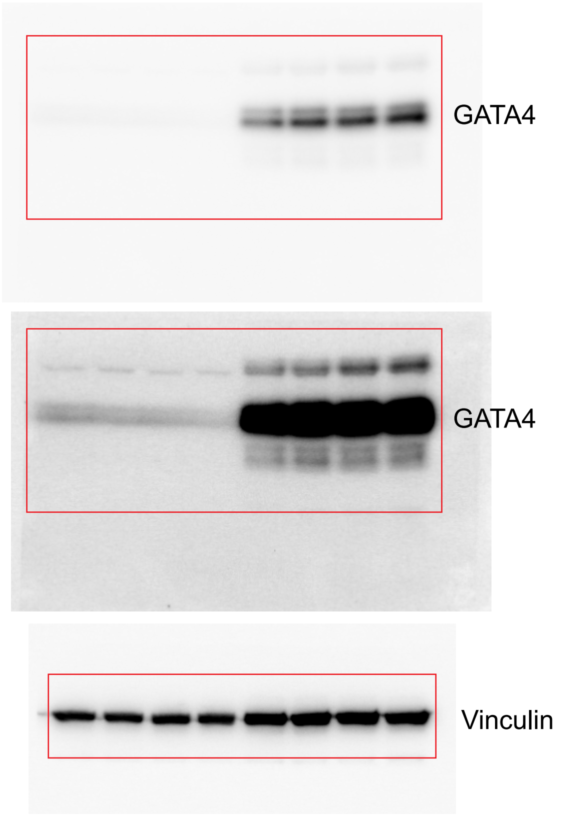

Supplementary Figure S7D

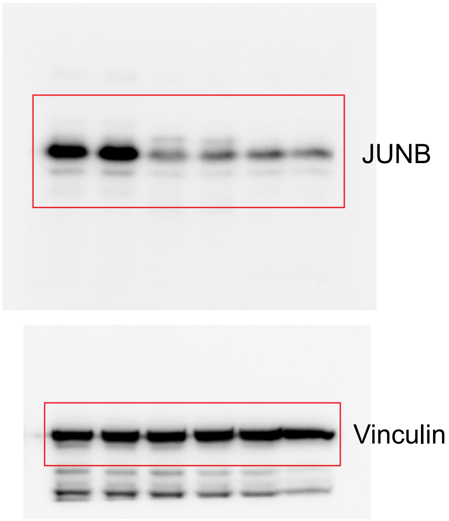

Supplementary Figure S5A

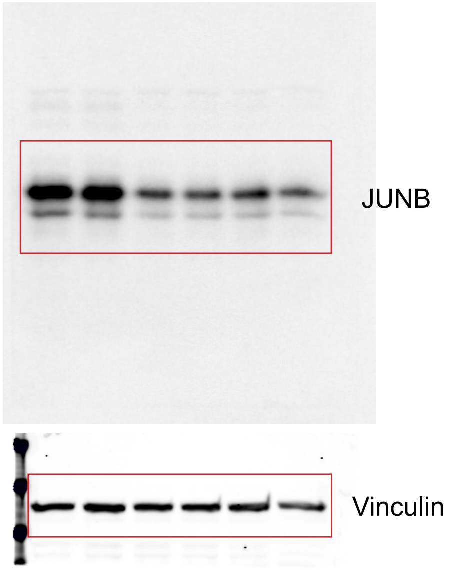

Supplementary Figure S8E

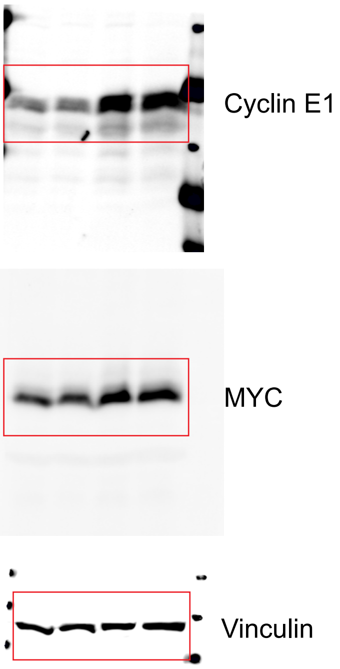

Supplementary Figure S8B

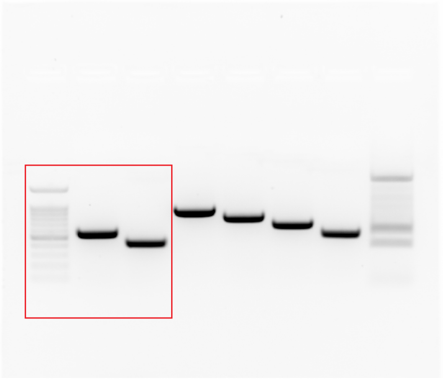

Supplementary Figure S9D

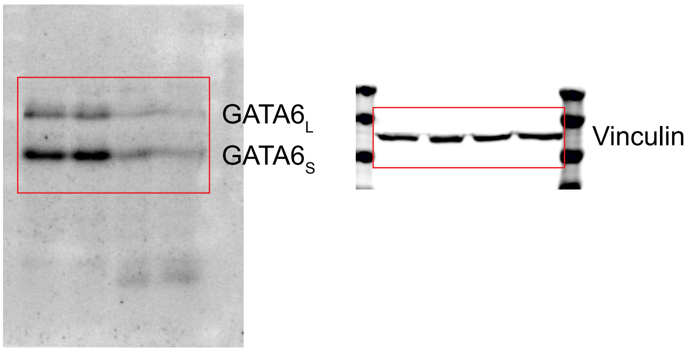

Supplement: Unedited blot and gel images [file jci-136-191370-s210.pdf]
